# A Practical Guide and Assessment on Using ChatGPT to Conduct Grounded Theory: Tutorial

**DOI:** 10.2196/70122

**Published:** 2025-05-14

**Authors:** Yongjie Yue, Dong Liu, Yilin Lv, Junyi Hao, Peixuan Cui

**Affiliations:** 1 School of Journalism and Communication Tsinghua University Beijing China; 2 School of Journalism and Communication Renmin University of China Beijing China; 3 Broadcasting and Anchoring School of Communication University of China Beijing China

**Keywords:** grounded theory, ChatGPT, manual coding, computer-assisted software, performance, human-AI collaboration

## Abstract

Generative large language models (LLMs), such as ChatGPT, have significant potential for qualitative data analysis. This paper aims to provide an early insight into how LLMs can enhance the efficiency of text coding and qualitative analysis, and evaluate their reliability. Using a dataset of semistructured interviews with blind gamers, this study provides a step-by-step tutorial on applying ChatGPT 4-Turbo to the grounded theory approach. The performance of ChatGPT 4-Turbo is evaluated by comparing its coding results with manual coding results assisted by qualitative analysis software. The results revealed that ChatGPT 4-Turbo and manual coding methods exhibited reliability in many aspects. The application of ChatGPT 4-Turbo in grounded theory enhanced the efficiency and diversity of coding and updated the overall grounded theory process. Compared with manual coding, ChatGPT showed shortcomings in depth, context, connections, and coding organization. Limitations and recommendations for applying artificial intelligence in qualitative research were also discussed.

## Introduction

The emergence of generative artificial intelligence, such as ChatGPT, has transformed academic research. Since its release by OpenAI in November 2022, ChatGPT has gained recognition for its ability to process and analyze natural language efficiently [1], making it valuable for tasks like thematic analysis, data extraction, and qualitative research [2,3]. However, its role in grounded theory analysis has not yet been investigated.

Grounded theory is widely used in health research that systematically develops theoretical insights through open, axial, and selective coding, making it well-suited for ChatGPT [4]. A review of studies from 2022 to May 2024 identified 8 empirical works using ChatGPT for qualitative research (details are provided in Multimedia Appendix 1), highlighting ChatGPT’s ability to generate coding results similar to those of manual approaches while enhancing efficiency and scalability [5-7]. However, limitations remain, particularly in capturing the contextual depth and ensuring consistency, which still requires human assistance [3,6,8]. Despite these challenges, ChatGPT can frequently adapt to the iterative and reflexive nature of grounded theory coding, even without training datasets [8]. Furthermore, ChatGPT can help explore different perspectives, aiding in theoretical framework development [3]. Nevertheless, there is no recent paper systematically exploring the application and effectiveness of ChatGPT in grounded theory, especially within the Chinese context.

Reliability is a key metric for evaluating ChatGPT’s coding performance, assessing whether different coders (human or machine) produce similar results [9-11]. Typical measures include percent agreement (comparing human and artificial intelligence [AI] coding similarity) and the κ coefficient (measuring agreement beyond chance) [12,13].

Based on the above, this study investigates ChatGPT’s application and performance in grounded theory using interview data from players who are blind in Listen and Play in Jianghu, a popular Chinese MMORPG designed for individuals who are blind. Specifically, this study aims to (1) provide detailed guidelines and practices for using ChatGPT in grounded theory within the Chinese context, (2) evaluate the effectiveness of ChatGPT coding by systematically comparing human coding with computer-assisted software to ChatGPT-assisted coding, and (3) explore the broader implications and future directions of ChatGPT in qualitative research.

## Methods

### Data Materials

From February to March 2022, we conducted semistructured web-based interviews with 8 participants from the Listen and Play in Jianghu game community and special education schools, aged 18 to 41 years. The interviews explored players’ gaming experiences and their impact on daily life, mental health, and overall well-being. Each interview was recorded and transcribed, and the results were a 40,000-word dataset. For data analysis, different researchers performed manual coding analysis with computer-assisted software and ChatGPT 4-Turbo coding analysis.

### Ethical Considerations

Ethics approval was obtained from the Research Ethics Committee of the School of Journalism and Communication, Renmin University of China (20220028). All participants provided informed consent and agreed to the secondary academic use of the data without the need for additional consent. The consent process included information about the purpose of the study, data usage, and participants’ rights, including their ability to withdraw at any time. All data were anonymized, and identifying information was removed during transcription, analysis, and reporting. No images or identifiable materials were used in the article or supplementary materials. Each participant received a compensation of 50 RMB (approximately US $6.86) for their participation.

### Manual Analysis With Computer-Assisted Software for Grounded Theory

For the first analytical approach, 2 researchers used the qualitative data analysis software NVivo (version 20; QSR International) to conduct 3-stage coding. They independently coded 3 randomly selected transcripts, resolving disagreements through discussion. Once intercoder reliability exceeded 85%, they applied the coding scheme to the remaining 5 interviews (simplified workflow is provided in Multimedia Appendix 2).

During open coding, meaningful concepts were identified and categorized. Through axial coding, these concepts were grouped into 8 main categories based on their relationships. Selective coding established “gaming motivations and impact” as the core category, linking and refining related concepts to develop a preliminary theoretical framework. Researchers iteratively refined this framework using a primary relationship diagram until it reached a stable form (coding results are provided in Multimedia Appendix 3).

### ChatGPT 4-Turbo Coding Analysis for Grounded Theory

In this section, we showcase the detailed procedure of another researcher using ChatGPT 4-Turbo for grounded theory coding analysis (simplified workflow is provided in Multimedia Appendix 2).

#### Step 1: Setting Up Background Prompts

**Figure 1:**
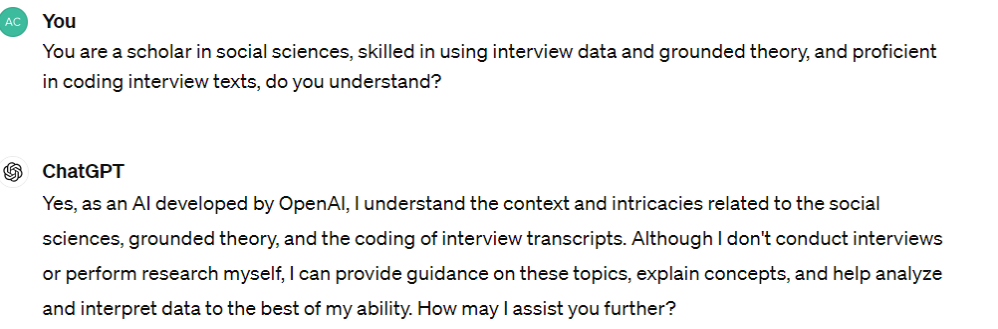
Prompt-response example from ChatGPT 4-Turbo.

**Prompt 1.1:***You are a scholar in social sciences, skilled in using interview data and grounded theory, and proficient in coding interview texts. Do you understand?* (See an example in Figure 1)

#### Step 2: Performing Open Coding

ChatGPT 4-Turbo has a context length of 8192 tokens, with each token typically representing about 4 characters. For Chinese text, one character corresponds to approximately 2 to 2.5 tokens [14]. Due to this token limit, shorter texts can be processed in a single entry, while longer texts exceeding the limit must be input incrementally.

Here are the prompts for inputting longer text lengths:

**Prompt 2.1:** *This is the first part of the interview text with person two, who is blind, about playing video games. Have you received it? You only need to remember the content of the first part and wait until all the text is sent before analyzing it.*

Person 2 who is blind:

Interviewer: Why did you start playing this game in the first place?... (insert full interview text here)

**Prompt 2.2:** *This is the second part of the interview text with person two, who is blind, about playing video games. Have you received it? (insert full interview text here)*

**Prompt 2.3:** *Please conduct open coding for this interview text with the “person 2 who is blind” (in two parts), and align the coding with the source in the text, presenting it in a table.*

However, we can input it all simultaneously when the text is not too lengthy. In this case, the prompt is as follows:

**Prompt 2.4:** *Now, you have received the interview text about person 1 who is blind, person 2 who is blind, and person 3 who is blind. Please conduct open coding for the interviews of person 1 who is blind, person 2 who is blind, and person 3 who is blind, and align each code with all specific statements in the text. One code can correspond to multiple statement sources. Please present the codes in a table. The table should have four columns: one for the code, one for the statement source (the sentence is required), one for the number of sources corresponding to that code, and one for the interviewees. (insert full interview text here)*

Whether the coding results are from long or short interview texts, if we feel that the coding results are not detailed enough, or if we want to obtain more coding results, we enter the following prompt words.

**Prompt 2.5:** *The coding above is not detailed enough; please delve deeper and identify more codes.*

Through the above steps, we obtain coding results from multiple perspectives. Afterward, we manually screen and merge these results to determine the final open coding outcomes.

#### Step 3: Performing Axial Coding

During axial coding, ChatGPT 4-Turbo was instructed to compare all encodings, identifying their differences and similarities and establishing relationships between primary and secondary categories. Additionally, we can instruct ChatGPT 4-Turbo to reassemble and cluster the text obtained from open coding in novel ways, seeking new categorical combinations. Significantly, ChatGPT 4-Turbo struggles to perform axial coding for 8 blind-person interviews simultaneously due to the limited number of tokens. We could conduct this in stages. Here are the prompts and operational procedure.

**Prompt 3.1:** *Here are the results of open coding for players 1 and 2. Can you compare the similarities and differences in all the encodings of person 1 who is blind and person 2 who is blind, and identify the themes they share as well as their unique themes? (insert full open coding results of person 1 and person 2).*

**Prompt 3.2:** *Can you categorize all encodings for persons 1 and 2 who are blind under appropriate themes? Each encoding can fall under multiple themes, and each theme might have sub-themes. Please list all theme classifications.*

**Prompt 3.3:** *Using a textual diagram, could you illustrate the relationships between these themes and their specific encodings?*

To identify more themes, we can use ChatGPT 4-Turbo for clustering analysis to identify new primary and secondary themes. This analysis can guide axial encoding and final selective encoding. The coding results might differ from previous theme classifications, potentially leading to new thematic categories. Below are the steps:

**Prompt 3.4:** *Please cluster all open coding nodes from interviews with persons 1 and 2 who are blind.*

**Prompt 3.5:** *I’d like you to forget the previous thematic analysis you’ve done. Using all open encodings from persons 1 and 2 who are blind, perform clustering again.*

Through these procedures, we identify primary and secondary themes about persons 1 and 2 who are blind. A manual review then refines and merges themes for more comprehensive axial coding.

Repeating this process, we obtain axial coding results for persons 3-4, 5-6, and 7-8, ultimately integrating the data from all 8 individuals. The prompt words are as follows:

**Prompt 3.6:** *There are axial coding results from a total of 8 individuals who are blind. This is the first part: (insert full axial coding results for individuals 1, 2, 3, and 4 who are blind). There will also be content from the second part. Please note that the content does not need to be analyzed.*

**Prompt 3.7:** *There are axial coding results from a total of 8 individuals who are blind. This is the second part: (insert full axial coding results for individuals 5 and 6 who are blind). There will also be content from the third part. Please note that the content does not need to be analyzed.*

**Prompt 3.8:** *There are axial coding results from a total of 8 individuals who are blind. This is the third part: (insert complete axial coding for individuals 7, and 8 who are blind). Please note that the content does not need to be analyzed.*

**Prompt 3.9:** *You have received all axial coding results from a total of 8 individuals who are blind. Can you compare the similarities and differences in all the encodings of person 1 to person 8, and identify the themes they share as well as their unique themes?*

**Prompt 3.10:** *Can you categorize all encodings for persons 1 to 8 who are blind under appropriate themes? Each encoding can fall under multiple themes, and each theme might have sub-themes. Please list all theme classifications and the corresponding interviewees.*

To explore additional themes and subthemes, we can re-input prompt words 3.4 and 3.5, then manually refine and merge the data to finalize the axial coding results.

#### Step 4: Performing Selective Coding

ChatGPT 4-Turbo is instructed to analyze relationships from axial coding, highlighting the dominant category [4] and linking it to others for a cohesive narrative structure [15]. Below are the prompts and operational procedures for this phase.

**Prompt 4.1:** *Please perform selective coding based on the following coded main categories and subcategories, identify the core category, and establish relationships around the core category. (insert full axial coding results)*

If we want to derive a range of selective coding outcomes, we can replicate the procedure in prompt 4.1, establishing diverse connections between the core and other categories. We then manually refined the data to finalize the selective coding results.

According to the core category of selective coding from ChatGPT 4-Turbo, we manually confirm the validity of the relationships between the core category and others, and then form a primary relationship diagram between the categories. Researchers returned to the initial interviews to find information and refine the theory, eventually creating a complete theoretical framework (the full ChatGPT coding results and generic prompts are provided in Multimedia Appendix 3).

### Analytic Approach

We used a systematic approach to comparing manual coding with computer-assisted software and ChatGPT 4-Turbo outputs. The interview texts were divided into 4 parts: persons 1 and 2, persons 3 and 4, persons 5 and 6, and persons 7 and 8 to facilitate the comparison and match the input order in the ChatGPT coding.

For open coding results, we first described the nodes, reference points, and coverage rate using numbers and percentages, then compared them through *t* tests in SPSS (version 26; IBM Corp). Next, we conducted a reliability check using NVivo (version 20; QSR International). To ensure consistency, we performed a semantic comparison between manually coded and machine-coded data, aiming to align all open codes [3]. This comparison was based on two main criteria: (1) the node names and category names generated by ChatGPT 4-Turbo coding are similar to those generated by manual coding assisted by computer software, and (2) the interview texts corresponding to the nodes coded by ChatGPT 4-Turbo and the descriptions of the categories convey a very similar meaning to those generated by manual coding assisted by computer software [3]. Initially, we invited one expert in the related field and 2 researchers to inspect and determine whether the node names and texts coded by both methods were semantically consistent. When a consistent meaning was observed across both methods, we aligned the language expression and calculated its inherent consistency. For example, the codes “people who are blind have limited daily activities” and “blind people's daily activities are restricted” have a similar meaning and can be aligned. We conducted checks and alignments on 274 ChatGPT 4-Turbo open codes and 289 manual open codes. Following this semantic alignment, we calculated the percentage of consistent coding and the κ coefficient. The κ coefficient ranges from –1 (complete disagreement) to 0 (random agreement) to +1 (complete agreement), with the following interpretations: 0.00-0.20 indicates slight agreement, 0.21-0.40 indicates fair agreement, 0.41-0.60 indicates moderate agreement, 0.61-0.80 indicates substantial agreement, and 0.81-1.00 indicates almost perfect agreement [16].

For axial and selective coding results, the expert and 2 researchers similarly conducted semantic comparisons and compared the relationships between categories to evaluate the similarities and differences between manual coding and machine coding, as well as the performance of ChatGPT 4-Turbo.

### Results of ChatGPT 4-Turbo Coding Performance

#### Open Coding

As shown in Table 1, manual open coding produced slightly more nodes than ChatGPT 4-Turbo (289 vs 274) and more reference points (333 vs 301), but neither difference was statistically significant (*P*>.05). Although manual open coding showed a higher coverage rate across all parts, the difference remained nonsignificant (*P*>.05). Additionally, manual coding yielded more reference points than nodes, whereas ChatGPT 4-Turbo produced nearly identical numbers for both. This is possible because ChatGPT 4-Turbo cannot merge reference points to specific nodes compared with humans.

**Table 1:** Performance of the manual open coding and ChatGPT 4-Turbo open coding.

			Persons 1 and 2		Persons 3 and 4		Persons 5 and 6		Persons 7 and 8		*P* value	
**Nodes,** **n (%)**												.87
	ChatGPT (n=274)	40 (14.60)		75 (27.37)		84 (30.66)		75 (27.37)				
	Manual (n=289)	47 (16.26)		67 (23.18)		85 (29.41)		90 (31.14)				
**Reference points,** **n (%)**												.90
	ChatGPT (n=301)	41 (13.62)		77 (25.58)		86 (28.57)		97 (32.23)				
	Manual (n=333)	54 (16.22)		76 (22.82)		96 (28.83)		107 (32.13)				
**Coverage rate** **(%)**												.60
	ChatGPT	35.32		45.27		35.73		44.64				
	Manual	56.37		46.92		36.70		51.39				

**Figure 2:**
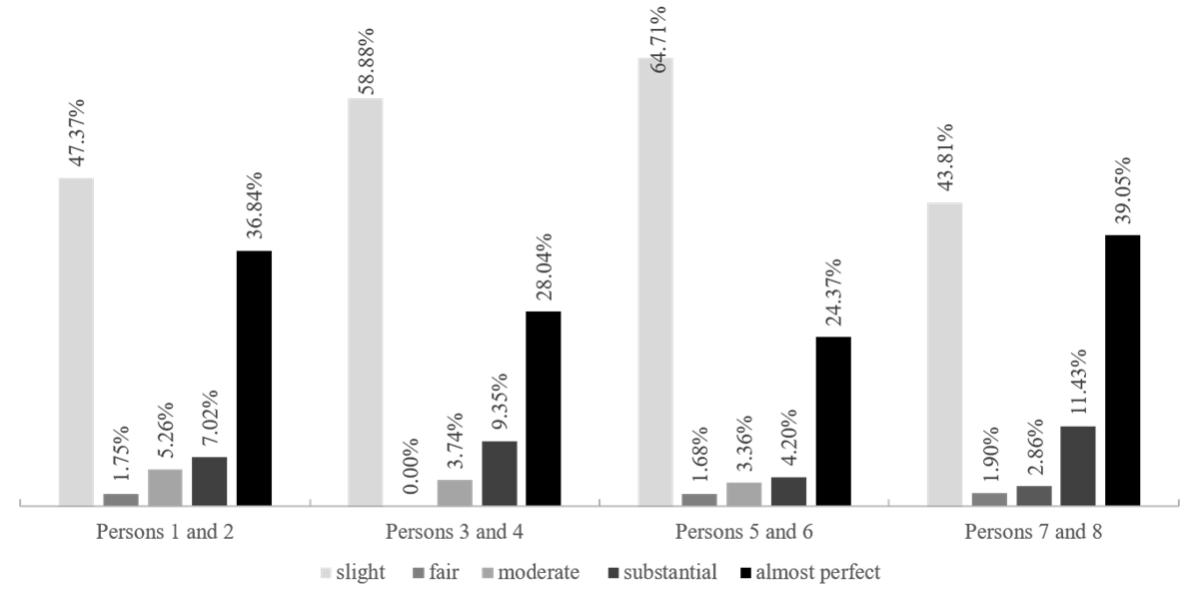
The distributions of kappa values in open coding of interview texts.

The percentage agreement between manual and ChatGPT 4-Turbo open coding was consistently above 95%, likely because a large portion of the text remained uncoded, leading to a high agreement rate. The κ coefficient distributions, as shown in Figure 2, followed a consistent pattern across text segments. “Slight agreement” constituted the highest proportion in every section, followed closely by “almost perfect agreement.” The proportion of “fair agreement” was the least among all categories.

#### Axial Coding

Manual coding identified 8 main categories, including “Interviewee’s personal information and background,” “Gaming motivation,” “Gaming behavior and process,” “Gaming impact,” “Gaming cognition and attitude,” “Gaming operational characteristics and accessibility design,” and “Gaming social interaction”; ChatGPT 4-Turbo coding produced 7 main categories, namely “Game motivation and attitude,” “Game experience,” “Game spending and investment,” “Impact of games on life,” “Game operation and learning,” “Social interaction in games,” “Game accessibility and design,” and “Personal information and background.” By comparing the names and semantics of the main categories and subcategories, we had the following findings: First, both analysis approaches produced 4 similar main categories: game impact, game interaction, game operation, and interviewee personal information. Second, manual axial coding generated categories and subcategories more generally and broadly, covering a wide range of topics related to “Gaming behavior and process” and “Gaming cognition and attitude.” Third, ChatGPT 4-Turbo axial coding offered a broader categorization that focuses on “Game experience” and “Game spending and investment.” Fourth, compared with manual axial coding, ChatGPT axial coding showed weaker capabilities in the connections and organization between main and subcategories.

#### Selective Coding

Researchers identified “gaming motivations and impact” as the core category and integrated it with other categories to develop the theoretical framework of “the motivation and impact mechanism for blind individuals” ChatGPT 4-Turbo selected “the impact of gaming on life” as the core category and explained its connections with other categories. Based on ChatGPT 4-Turbo’s textual explanations, researchers combined interview data to develop a theoretical framework, namely “the impact mechanism of gaming on life for blind individuals.” In this framework, the gaming motivation of blind individuals still played a crucial role. By conducting semantic comparisons of the categories and comparing their relationships, we discovered that the theoretical framework constructed through the 2 approaches shared many similarities. However, ChatGPT also fell short in depth of interpretation, understanding of context, and discerning subtle differences.

## Discussion

### Summary

This study compared the performance of manual coding with computer-assisted software and ChatGPT 4-Turbo coding across various metrics. It also provided detailed guidance on integrating ChatGPT 4-Turbo into grounded theory analysis. Serving both as a practical tutorial and an innovative contribution to qualitative studies, this study demonstrated the feasibility and increased efficiency of ChatGPT 4-Turbo in grounded theory, offering researchers insights into its application. Moreover, our findings revealed that both ChatGPT 4-Turbo coding and manual coding with computer-assisted software exhibited reliability in many aspects. Notably, ChatGPT 4-Turbo coding also enhanced the diversity and efficiency of coding, which is sometimes not obvious and difficult for humans. However, it struggled with depth, context, subtle nuances, connections, and coding organization.

First and foremost, this study underscores the potential of ChatGPT 4-Turbo in grounded theory. The pivotal role of language in the coding process and the selection of specific codes within grounded theory is well-known, and ChatGPT 4-Turbo excels in identifying, comprehending, and generating human-like text [1]. Our 3-step coding process with ChatGPT 4-Turbo—open, axial, and selective coding—mirrors manual coding, highlighting the interaction between human or AI coders and the text. This systematic, inductive analysis shows a strong consistency between the 2 approaches in terms of node, reference points, coverage rate, and reliability. These findings suggest that ChatGPT 4-Turbo could be a reliable and efficient tool for qualitative data analysis, sparking new possibilities in grounded theory.

Second, this study identifies key differences between human and ChatGPT 4-Turbo coding processes. Manual coding with computer-assisted software is iterative and evolves. As Glaser and Strauss [17] described, human coders must revisit the data repeatedly, continually draw comparisons, and often return to the primary data for further contrast and insights. Each iteration deepens their understanding, which is promptly documented and then informs subsequent coding phases. This approach is deeply rooted in personal experiences and contextual interpretations, requiring a perpetually open and fluid method toward the data [18]. In contrast, ChatGPT 4-Turbo relies on a large language model (LLM) and a multistep inferential process. When provided with clear coding prompts and interview transcripts, ChatGPT 4-Turbo uses its training data to forecast the most plausible responses and generate appropriate coding outputs [8]. Users can refine results through repeated inputs and aggregation. Hence, the entire ChatGPT 4-Turbo coding process is distinctly aggregative and structured.

Third, performance differences exist between manual and ChatGPT 4-Turbo coding. Specifically, while both approaches yield more reference points than nodes, manual open coding produces a significantly higher number of reference points relative to nodes than ChatGPT 4-Turbo. This aligns with previous research showing that when deeply immersed in the topic, human coders can merge reference points under broader thematic nodes based on nuanced understanding [18]. In contrast, ChatGPT’s algorithmic approach may limit such nuanced merging, which is consistent with findings on LLM’s challenges in capturing textual depth and interconnectedness [3]. Besides, due to ChatGPT 4-Turbo's text input length limitation, users have to split long texts and input them sequentially for encoding, which can result in some detailed content not being merged. Furthermore, reliability trends show high agreement between ChatGPT 4-Turbo and human coders at the sentence level. ChatGPT 4-Turbo also generated many open coding results that differ from those produced manually, indicating the diversity and richness of machine coding. During the axial coding process, both methods also generated similar and different categories, though manual coding demonstrated stronger connections and organization. In selective coding, while core category names differed, the overall narrative meaning and theoretical construction remained similar. Consistent with prior studies, ChatGPT shows weaknesses in depth, context, and subtle nuances [19].

Fourth, consistent with prior research [8,20], our findings indicate that ChatGPT 4-Turbo sometimes struggles to recognize specific textual information or provides incorrect encoding, particularly with lengthy or logically complex paragraphs. This can lead to overlooking crucial information, resulting in wrong encoding. For example, the statement “I did not play the game much afterward, and essentially in our group, I always ranked last” was encoded as “A decline in self-assessment due to game rankings.” This phenomenon, often called a “hallucination,” may stem from 2 key factors. On the one hand, each response depends on the ongoing context, including previous responses to those queries. Essentially, the software continuously attempts to predict what will happen next, meaning any error in its responses can be amplified in subsequent interactions [5]. On the other hand, biases inherent in the training dataset may reinforce stereotypes while overlooking the gaming experiences of individuals who are blind [21], causing the model to generate inaccurate or misleading interpretations to fill in gaps. Hence, researchers must be familiar with the data when using ChatGPT for analysis and to correct inaccuracies effectively.

### Implications for Qualitative Research

ChatGPT 4-Turbo coding significantly accelerates the coding process compared with traditional manual methods. This study took 2 researchers 3 weeks to complete the manual coding with computer-assisted software, whereas ChatGPT 4-Turbo finished the process in just one day after the prompts were confirmed. Historically, the relatively low proportion of qualitative research in published studies has been primarily due to the inefficiency of manual coding [22]. The efficiency of AI-driven coding frees up valuable time for researchers to focus on comprehensive analysis, which may increase the volume of qualitative studies and enrich the field with deeper insights and diverse perspectives. In health research, this could support faster and more robust analysis of interviews with vulnerable populations and public health data, thereby informing timely interventions and policy decisions.

Beyond speed, manual and AI-driven coding differ in uncovering textual meanings. Manual coding can reveal deep, intrinsic meanings underlying texts. In contrast, AI-driven coding, such as ChatGPT 4-Turbo, excels at detecting patterns not immediately apparent to human researchers but may overlook subtle nuances and interpretive categories [5,23]. Additionally, AI-driven coding introduces biases, including datasets, algorithms, cultural, and linguistic bias [21]. These biases can distort interpretations, potentially leading to harmful or misleading content [21]. Given these considerations, augmented qualitative research is encouraged, referring to the integration of AI’s efficiency with human expertise to ensure qualitative rigor while mitigating biases [23]. This is particularly critical in health contexts, where misinterpretation of qualitative data could affect vulnerable populations or contribute to inaccurate health outcomes.

As AI technologies advance, their accuracy is expected to improve, reducing errors and refining interpretations [18]. This transformation is likely to extend to qualitative analysis software, and we anticipate the emergence of integrated software with AI auto-coding functionalities shortly. However, this advancement brings ethical considerations, such as data privacy and algorithmic bias [1]. Addressing these challenges requires collaboration between qualitative researchers and computational experts to ensure AI is used responsibly and effectively in qualitative inquiry.

### Limitations and Future Directions

This study is not without limitations. First, this study is a small-sample exploratory study with only 8 interviewees. Future research should expand the sample size and incorporate diverse types across different domains to assess the application of AI-driven coding. Additionally, researchers could explore how ChatGPT 4-Turbo performs in large-scale datasets compared with manual coding and other AI models. One potential approach is to use LLMs combined with RAG (retrieval-augmented generation) to efficiently extract potential themes or preliminary categories from big data, followed by human review and refinement to establish a reliable analytical framework. Using this refined framework, RAG-enhanced LLMs can be used again for extraction and analysis. Comparative studies analyzing AI efficiency, accuracy, and bias across different dataset sizes would provide valuable insights into scalability and reliability in the future. Second, this study provides a guide to a single AI tool—ChatGPT 4-Turbo—for grounded theory. AI technologies, including ChatGPT 4-Turbo, have inherent biases and instability that may lead to confusion and incorrect coding outcomes. As AI technologies evolve, future studies should systematically evaluate AI-driven coding methodologies across disciplines. Longitudinal studies could track AI-generated coding over time, measuring consistency and identifying trends in its interpretive strengths and weaknesses. Future studies could also involve 2 or more LLMs independently coding the data, followed by manual organization and cross-validation, to enhance the stability and reduce biases in the coding results. Third, this study is limited to the Chinese language and context. Future research could extend the AI-driven coding methodology to larger global geographies and various ethnic contexts, examining how language-specific nuances and cultural factors impact the performance of AI models.

## Appendix

Multimedia Appendix 1 A review of ChatGPT’s application in qualitative research.

Multimedia Appendix 2 Simplified workflow.

Multimedia Appendix 3 Full coding results, theroretical model, and generic prompt.

## Abbreviations

AI: artificial intelligence
LLM: large language model
