# Supplementary material for: A Practical Guide and Assessment on Using ChatGPT to Conduct Grounded Theory: Tutorial
Source: J Med Internet Res. 2025 May 14;27:e70122. doi: 10.2196/70122 (PMC12120365; doi:10.2196/70122)
Supplement: Multimedia Appendix 1 [file jmir_v27i1e70122_app1.docx]

**Appendix 1. A Review of ChatGPT’s Application in Qualitative Research**

Table S1. The main characteristics of related research.

| **Authors** | **Qualitative analysis** | **Language** | **Dataset** | **Large Language Model** | | **Comparison between ChatGPT and human analytic results** |
| --- | --- | --- | --- | --- | --- | --- |
| Hamilton et al. [1] | Thematic analysis | English | Interview text | | ChatGPT | There is some overlap in about half of the human-centered themes and 80% of the ChatGPT themes. |
| Jalali and Akhavan [2] | Text analysis | English | Semi-structured interviews | | ChatGPT (4-Turbo) | ChatGPT identified mechanisms similar to those found by humans, as well as mechanisms that humans had not discovered. ChatGPT did not identify all the nuances that humans recognized. |
| Mesec [3] | Text analysis | English | Interview text | | ChatGPT | ChatGPT provided more specific and surprisingly appropriate answers. Humans provided more abstract answers. |
| Morgan [4] | Thematic analysis | English | Focus group interview text | | ChatGPT | ChatGPT did a very similar job to humans in capturing the key themes. |
| Paoli [5] | Inductive thematic analysis | English | Semi-structured interviews | | ChatGPT (3.5-Turbo) | ChatGPT identified most themes similar to those recognized by humans and showed a good degree of validity. |
| Siiman et al. [6] | Deductive analysis | English | Chats text | | ChatGPT (4-Turbo) | The results from ChatGPT and human results show substantial agreement (kappa from 0.706 to 0.782). |
| Siiman et al. [6] | Inductive analysis | English | Chats text | | ChatGPT (4-Turbo) | A moderate positive correlation was found between AI-generated scores and human-coded scores (r=0.621). |
| Xiao et al. [7] | Deductive coding analysis | French & English | Children’s curiosity-driven questions | | ChatGPT (3-Turbo) | ChatGPT achieved fair to substantial agreements with expert-coded results. |
| Perkins and Roe [8] | Inductive thematic analysis | English | Website content | | ChatGPT (4-Turbo) | NA(comparisons of the analysis results are not presented) |

Table S2. Advantages and disadvantages of applying ChatGPT in qualitative analysis in related research.

| **Authors** | **Advantages of using LLMs for analysis** | **Disadvantages of using LLMs for analysis** |
| --- | --- | --- |
| Hamilton et al. [1] | 1. Enable efficient and speedy data processing. 2. Identify hidden patterns and themes. uncovering new insights. 3. Analyze extensive datasets. | 1. Lack of current events awareness due to no real-time internet access. 2. Lack of transparency in theme generation criteria. 3. Fail to grasp depth, context, and interpretive nuances. |
| Jalali and Akhavan [2] | 1. Recognize details, enriching analysis. 2. Enhance objective and straightforward analysis. | 1. Lack of nuanced understanding and integration with broader theories. 2. Misinterpret causal relationships. 3. Raise ethical and data ownership concerns. 4. Fail to yield consistent output. 5. Introduce biases during analysis. |
| Mesec [3] | 1. Creatively paraphrase statements. 2. Infer implied meanings. | 1. Repeat unproductively. 2. Add inappropriate terms. 3. Misunderstand questions. 4. Overlook relevant statements. |
| Morgan [4] | 1. Simplify qualitative data analysis. 2. Save significant time compared to manual coding. | 1. Emphasize specifics over the bigger picture. 2. Mismatch with intensely inductive approaches. |
| Paoli [5] | 1. Identify overlooked codes and patterns, and enhance qualitative analysis. 2. Generate diverse and rich themes. | 1. Generate responses based on probability, sometimes inconsistent. 2. Need to be fully anonymized during analysis. 3. Produce hallucinations sometimes. |
| Siiman et al. [6] | 1. Enhance transparency in coding data. 2. Analyze extensive datasets. | 1. Lead to bias inadvertently. 2. Designing useful prompts for specific contexts is difficult. |
| Siiman et al. [6] | 1. Enhance transparency in coding data. 2. Analyze extensive datasets. | 1. Lead to bias inadvertently. 2. Designing useful prompts for specific contexts is difficult. |
| Xiao et al. [7] | 1. Analyze extensive datasets. | 1. Produce incorrect labels. |
| Perkins and Roe [8] | 1. Increased objectivity. 2. Increased efficiency. 3. Increased cognitive support for the researcher. | 1. Limit handling of complex datasets. 2. Require additional time to develop necessary GenAI-related skills. |

**Reference**

1. Hamilton L, Elliott D, Quick A, Smith S, Choplin V. Exploring the Use of AI in Qualitative Analysis: A Comparative Study of Guaranteed Income Data. *Int J Qual Methods*. 2023;22:16094069231201504. [doi:10.1177/16094069231201504]

2. Jalali MS, Akhavan A. Integrating AI language models in qualitative research: Replicating interview data analysis with ChatGPT. *Syst Dyn Rev*. Published online May 21, 2024:sdr.1772. [doi:10.1002/sdr.1772]

3. Mesec B. The language model of artiﬁcial inteligence chatGPT - a tool of qualitative analysis of texts. *Authorea Preprints.* Published online April 18, 2023. URL:https://d197for5662m48.cloudfront.net/documents/publicationstatus/136160/preprint_pdf/0bf1ebc3f8369fda95b9e762cd65897b.pdf [accessed 2024-5-15]

4. Morgan DL. Exploring the Use of Artificial Intelligence for Qualitative Data Analysis: The Case of ChatGPT. *Int J Qual Methods*. 2023;22. [doi:10.1177/16094069231211248]

5. De Paoli S. Performing an Inductive Thematic Analysis of Semi-Structured Interviews With a Large Language Model: An Exploration and Provocation on the Limits of the Approach. *Soc Sci Comput Rev*. Published online December 7, 2023:08944393231220483. [doi:10.1177/08944393231220483]

6. Siiman LA, Rannastu-Avalos M, Pöysä-Tarhonen J, Häkkinen P, Pedaste M. Opportunities and challenges for AI-assisted qualitative data analysis: An example from collaborative problem-solving discourse data. In: Springer; 2023:87-96.

7. Xiao Z, Yuan X, Liao QV, Abdelghani R, Oudeyer PY. Supporting Qualitative Analysis with Large Language Models: Combining Codebook with GPT-3 for Deductive Coding. In: *28th International Conference on Intelligent User Interfaces*. ; 2023:75-78. [doi:10.1145/3581754.3584136]

8. Perkins M, Roe J. The use of Generative AI in qualitative analysis: Inductive thematic analysis with ChatGPT. *J Appl Learn Teach*. 2024;7(1). [doi:10.37074/jalt.2024.7.1.22]
