# Supplementary material for: A Practical Guide and Assessment on Using ChatGPT to Conduct Grounded Theory: Tutorial
Source: J Med Internet Res. 2025 May 14;27:e70122. doi: 10.2196/70122 (PMC12120365; doi:10.2196/70122)
Supplement: Multimedia Appendix 2 [file jmir_v27i1e70122_app2.docx]

**Appendix 2- Simplified workflow**
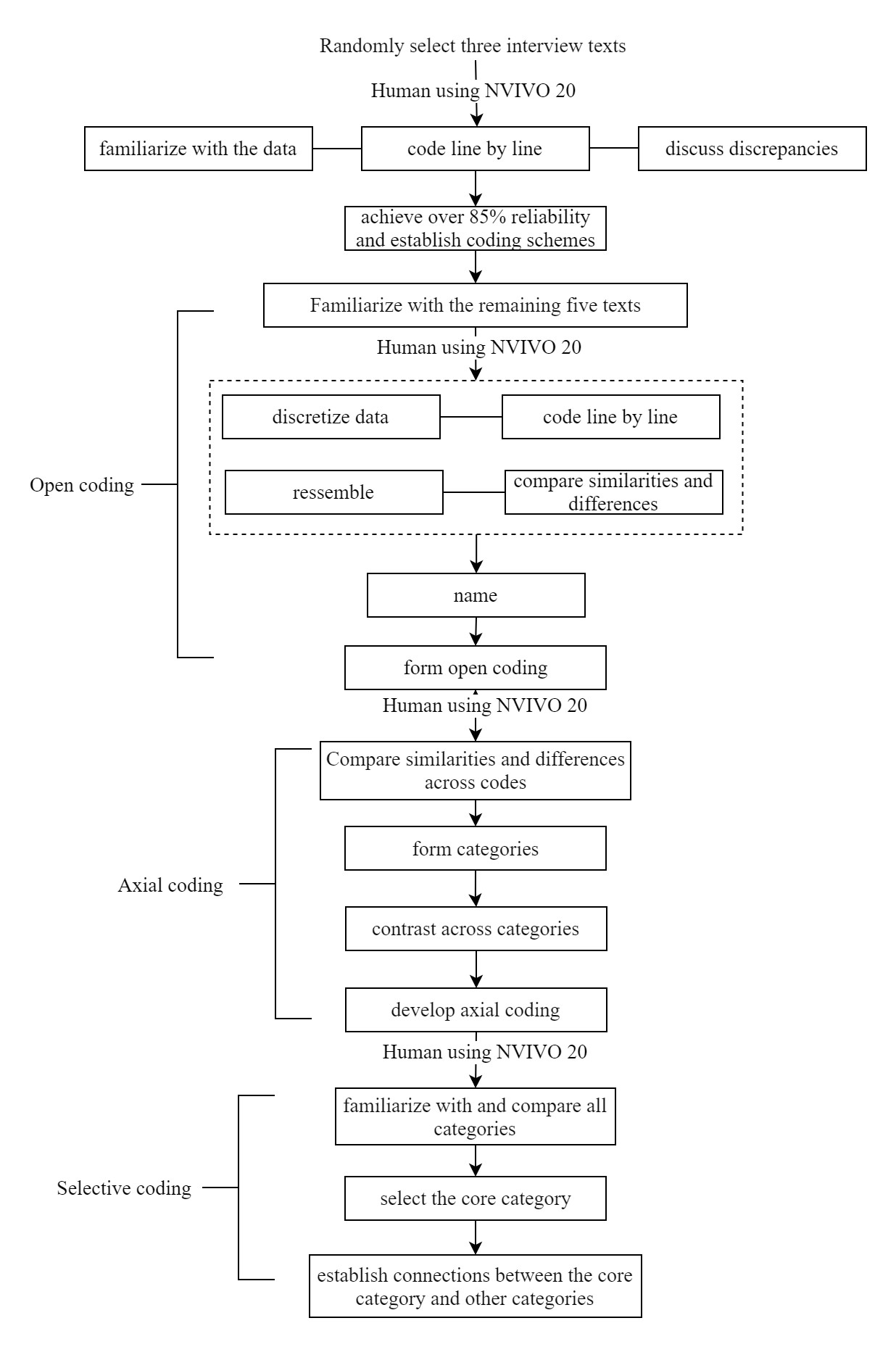


Figure S1. Simplified workflow for grounded theory with humans using NVIVO 20.


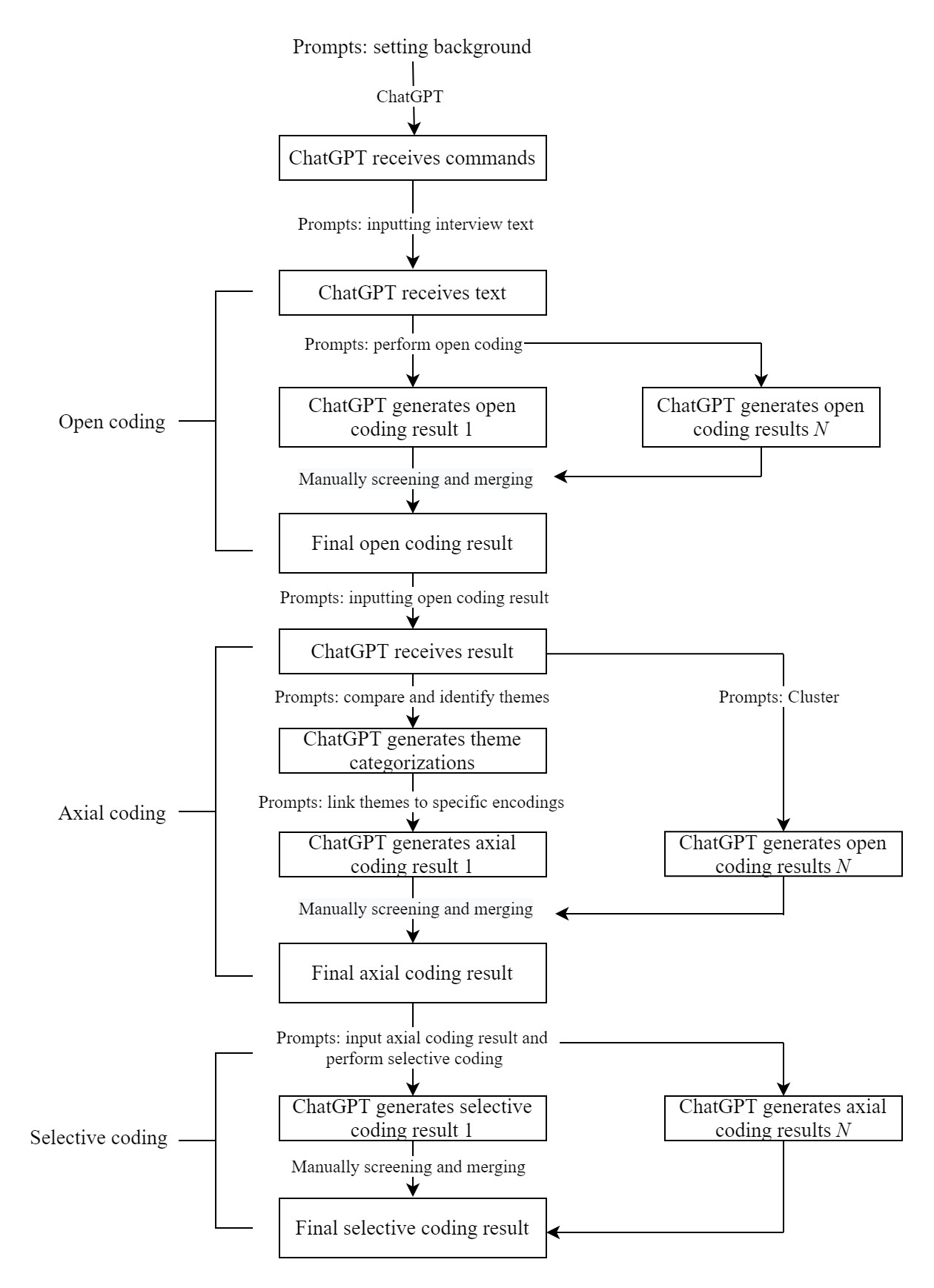


Figure S2. Simplified workflow for grounded theory using ChatGPT (4-Turbo).
