# Supplementary material for: A Practical Guide and Assessment on Using ChatGPT to Conduct Grounded Theory: Tutorial
Source: J Med Internet Res. 2025 May 14;27:e70122. doi: 10.2196/70122 (PMC12120365; doi:10.2196/70122)
Supplement: Multimedia Appendix 3 [file jmir_v27i1e70122_app3.docx]

**Appendix 3**

Contents

[I. ChatGPT (4-Turbo) open coding results 1](#_Toc171199342)

[Table S3. ChatGPT (4-Turbo) open coding results for blind person 1 1](#_Toc171199343)

[Table S4. ChatGPT (4-Turbo) open coding results for blind person 2 3](#_Toc171199344)

[Table S5. ChatGPT (4-Turbo) open coding results for blind person 3 5](#_Toc171199345)

[Table S6. ChatGPT (4-Turbo) open coding results for blind person 4 8](#_Toc171199346)

[Table S7. ChatGPT (4-Turbo) open coding results for blind person 5 10](#_Toc171199347)

[Table S8. ChatGPT (4-Turbo) open coding results for blind person 6 15](#_Toc171199348)

[Table S9. ChatGPT (4-Turbo) open coding results for blind person 7 17](#_Toc171199349)

[Table S10. ChatGPT (4-Turbo) open coding results for blind person 8 20](#_Toc171199350)

[II. ChatGPT (4-Turbo) axial coding results 24](#_Toc171199351)

[Table S11. ChatGPT (4-Turbo) axial coding results for blind person 1-2 24](#_Toc171199352)

[Table S12. ChatGPT (4-Turbo) axial coding results for blind person 3-4 27](#_Toc171199353)

[Table S13. ChatGPT (4-Turbo) axial coding results for blind person 5-6 30](#_Toc171199354)

[Table S14. ChatGPT (4-Turbo) axial coding results for blind person 7-8 34](#_Toc171199355)

[Table S15. ChatGPT (4-Turbo) axial coding results for blind person 1-8 39](#_Toc171199356)

[III. Manual axial coding results 41](#_Toc171199357)

[IV. ChatGPT (4-Turbo) selective coding results 43](#_Toc171199358)

[V. Theoretical model coded by manual coders 45](#_Toc171199359)

[VI. Theoretical model coded by ChatGPT (4-Turbo) 46](#_Toc171199360)

[VII. Generic prompts for grounded theory coding using ChatGPT (4-Turbo) 47](#_Toc171199361)

# ChatGPT (4-Turbo) open coding results

## Table S3. ChatGPT (4-Turbo) open coding results for blind person 1

| **Code** | **Text** |
| --- | --- |
| Limitations of blind people's daily activities | Blind person 1: There's not much that blind people can do on a daily basis, other than massages, and the rest of the time is spent playing games... |
| Games as a time-killing tool | Blind person 1: The rest of the time is just spent playing games... |
| Control over games | Blind person 1: And frankly, games are controlled by people; it's people who play games, not games that control us... |
| Understanding the goal of the game | Blind person 1: Don't go chasing after high combat power, good equipment, and those things in the game... |
| Attitude towards game consumption: No payment | Blind person 1: As long as you don't spend money, just enjoy it. |
| Favorite game segments: Imperial examination and drinking quiz | Blind person 1: My favorite parts are the imperial examination and the drinking quiz, where you can use your brain... |
| Views on in-game recharge | Blind person 1: Many people, including those I know, rely heavily on spending money in the game to improve combat power, level, and equipment, which I think is unnecessary. |
| Initial contact with the game: Introduced by a friend | Blind person 1: When I first came into contact with the game, it was a friend who took me there. |
| Original intention of the game: Self-entertainment, not controlled by the game | Blind person 1: I think my original intention hasn't changed, which is not to let the game play you, but to be in control as a person. |
| Games as a social tool | Blind person 1: Playing this game indeed allows you to meet a lot of friends, all kinds of people. |
| Attitude towards the discontinuity of the game | Blind person 1: There was a time when I didn't play this game for more than a year, but I logged in with my own mobile number, and I didn't want to sell it to others to play, so I just play when I have time, and stop when I don't. That's how it is. |
| Team composition preference: Acquaintances first | Blind person 1: I prefer to play with familiar teams in the game... |
| Views on the sense of achievement in the game | Blind person 1: For example, whether there is a sense of achievement in playing this game, although some people may have improved their combat power in this world, and seem invincible in this virtual world... But I don't think so... |
| Gains from the game: Diversified entertainment methods | Blind person 1: Actually, I feel that the biggest gain is that the game allows us blind people to have more entertainment methods and ways during our free time. |
| Suggestions for game improvement: Add more mini-games | Blind person 1: If there are more mini-games in the game, that would be good, this is my personal opinion. |

## Table S4. ChatGPT (4-Turbo) open coding results for blind person 2

| **Code** | **Text** |
| --- | --- |
| Initial gaming motivation: Killing time | Blind Man 2: I play to pass the time when I have nothing to do. |
| Initial gaming motivation: Socializing | Blind Man 2: Playing games allows me to meet many people... |
| Gaming duration: One and a half years | Blind Man 2: I've been playing for almost one and a half years... |
| Recharge amount: Over a hundred thousand | Blind Man 2: I've already recharged over a hundred thousand in the game... |
| Reflection after recharging: Wasteful | Blind Man 2: It's just a waste of money... |
| Change in attitude towards game investment: From recharging to regret | Blind Man 2: I regret it now and have stopped recharging. |
| Cognition of game's virtuality: Difference between game and reality | Blind Man 2: The game is virtual. |
| Game identity: Gang leader | Interviewer: How long have you been playing? You are now the gang leader? |
| Change in game ranking: From strong to weak | Blind Man 2: I'm now one of the weaker players. |
| Dissatisfaction with game status: Not as powerful due to not recharging | Blind Man 2: I'm not powerful anymore. |
| How the game was introduced: Introduced by a classmate | Blind Man 2: My classmate was playing, so I started playing too. |
| Social function of the game: Making friends | Blind Man 2: I've made many friends playing, so I can't let go. |
| Game's attraction: Realizing a fantasy dream | Blind Man 2: Everyone has a fantasy dream, everyone wants to be the number one in martial arts... |
| Source of game achievement: Becoming the top in martial arts | Blind Man 2: Everyone wants to be the number one in martial arts... |
| Source of game achievement: Satisfying vanity | Blind Man 2: To be honest, it's all about vanity. |
| Reason for staying in the game: Social relationships | Blind Man 2: I continue to play because I've met two really good friends in the game... |
| Negative evaluation of the game: Devil | Blind Man 2: So this game is also a devil. |
| Negative effects of the game: Excessive spending | Blind Man 2: Vanity costs money, so don't get too involved. |
| Pressure to recharge: Not to hold back friends | Blind Man 2: Another reason is because of your friends. If they improve and you don't, you'll hold them back. |
| Warning about game addiction: Devilish and addictive | Blind Man 2: This game is truly devilish and addictive... |
| Positive gains from the game: Broadened mindset | Blind Man 2: This game has broadened |
| Positive gains from the game: Judging character | Blind Man 2: Even though it's virtual, you can still judge a person's character from it... |
| Negative emotions from the game: Sadness from losing | Blind Man 2: Losing in the game can be very heartbreaking. |
| Human nature issues in the game: Revengeful behavior | Blind Man 2: As a result, he teamed up with other gangs to attack us, that's revenge... |
| Attitude towards the virtual world: No need to take virtual things too seriously | Blind Man 2: I later realized there's no need, there's no need to take virtual things too seriously... |

## Table S5. ChatGPT (4-Turbo) open coding results for blind person 3

| **Code** | **Text** |
| --- | --- |
| Game exposure history | I was introduced to this game quite early, but I stopped playing after reaching around level 40. |
| Reason for temporarily quitting | I stopped playing after reaching around level 40 and just left it. |
| Reason for resuming the game | Two years later, a master came to my shop. They played it well and invited me and another master to play together. |
| Official start time of the game | So I officially started playing around the beginning of 2019, and have been playing for over two years now. |
| Daily game duration | Now, I play the game daily, totaling about three hours. |
| Tedium due to excessive gaming | Because I played for too long, it became monotonous. Every day was the same, and it wasn't joyful anymore; it felt like a task. |
| Time of game interruption | So I took a break for about a month and a half. Later, when the tasks became simpler, I started playing again. |
| Total investment in the game | I've recharged a total of about 500 yuan, but later earned a few hundred more. |
| Joy from in-game economic activities | Selling items and making money in the game does bring moments of happiness. |
| Attitude towards in-game recharges | If you want to be powerful in the game, you have to recharge. I don't see the point in spending my own money on the game, so I only use the money I earn in-game. |
| Social motivation for gaming | I play this game to make like-minded friends, not just for entertainment. There are plenty of entertainment options available nowadays. |
| Reaction to friends quitting the game | If the friends I'm close to in the game quit, I would quit too. |
| Main social contacts in the game | Mainly, I play with friends. When they're busy, I'd invite others, expanding my circle of friends in the game. |
| Observation and attitude towards other players | After playing for so long, I've noticed many play the game to vent. Not everyone is suitable to be friends. |
| View on aggressive behavior in the game | Some people, when frustrated in real life, especially those who are introverted or facing challenges, would spend money in the game to attack others, finding joy in such aggressive behaviors. |
| Extension of in-game social activities to real life | My personal intention is to make friends through the game and transition from the virtual world to real life. That's why I created a game group. |
| Activities in the game group | In that group, we don't just talk about the game. We often discuss various aspects of life and work. |
| Improvement in life brought about by game socialization | When I face troubles or feel down in life, I can chat and communicate with them. They can guide and comfort me. |
| Economic help limit from game socialization | However, they can't provide much economic assistance. |
| Unprovoked attack experience | I was peacefully enjoying the hot springs in the game, and without provoking him, he attacked and kicked me out. |
| Power confrontation | I then used my other account to confront him, and I managed to kick him out. |
| Understanding of game character levels | I think I was around level 80, and that person was also in the 80s. |
| Continuous attacks encountered | He chased and attacked me every day, especially when I was enjoying the hot springs. |
| Oppression from powerful players | He had higher combat power and managed to kick me out. |
| Social support | Later, I met a skilled friend. We played and chatted together. He had a powerful account and stood up for me when he saw that person bullying me. |
| Sense of justice in the game | He told me to rest assured and inform him if that person attacked me again, and he would retaliate. |
| Defensive mentality in the game | But I couldn't defeat him, so I had to avoid him. |
| Revenge behavior in the game | He angered me, and in my frustration, I even spent money. |
| Cooperation and competition in the game | We later participated in gang wars together, verbally calling each other "brothers", but would fight upon meeting. |
| Healthy social boundaries | I impulsively deleted his WeChat, thinking we couldn't get along. |
| Psychological challenges in the game | I challenged him to a blood battle. My combat power was tens of thousands lower than his, but I wore internal defense while he wore external. I defeated him, costing him 70 to 80 points. |
| Negative emotions from the game | Mostly negative emotions, not much positivity. After all, it's just a game and quite draining. |
| Social value of the game | Apart from making friends, there's not much positivity. |
| Time consumption in the game | It's a way to pass the time when there's nothing else to do, making life a bit more fulfilling. |
| Brief happiness in the game | Snatching monsters and succeeding when others fail does bring a bit of happiness. |
| Economic gains in the game | Or when we sell items and make money, there are moments of happiness. |
| Self-awareness in the game | Initially, I felt frustrated and powerless when I couldn't defeat others. But on reflection, others spent money while I didn't, so it's natural. |
| Skills and strategies in the game | Another player and I are considered skilled. We didn't spend much money, but our combat power is relatively high. |
| Impact of the game on the sense of fulfillment in life | It does add some fulfillment, but it's also quite distracting. |
| Time consumption of the game | If you want to increase your combat power and find more joy in the game, you have to spend money and invest a lot of time. |
| Repetitiveness in the game | After playing for a long time, it becomes monotonous. Every day is the same, and it's no longer joyful. |
| Impact of the game on work | Sometimes the game affects work, so I try to control and reduce my playtime, ensuring it doesn't interfere with my job. |
| Social value in the game | The only advantage for me is making more friends, getting to know these good friends, which makes me happier. |
| Offline social possibilities brought by the game | Meeting offline hasn't happened yet. A few of us who play well together have already planned to meet offline and hang out. |
| Gift exchange and travel invitations in the game | A friend from Sichuan wanted to send me sausages, but I declined. Others invited me to travel and hang out. |
| Diverse topic exchanges in the game | We chat about various topics, not just the game, but also life and work. |
| The game as a bridge for socialization | Without the game, even if we chatted on WeChat, we wouldn't interact as much. In the game, we can do tasks together, participate in activities, and communicate more deeply, getting to know more people. |
| Broad geographical social contacts brought by the game | I like making friends. I've met people from Guangdong, Sichuan, Chongqing, Beijing, and many others on WeChat. |
| The game as a means to cope with loneliness | When feeling lonely, the game can be a way to pass the time and find some joy. |

## Table S6. ChatGPT (4-Turbo) open coding results for blind person 4

| **Code** | **Text** |
| --- | --- |
| Entering the game through social networks | "We have a friend. We have a good friend, and we both know a mutual friend. He introduced me." |
| Team collaboration in the game | "At that time, we had to form a team to clear the dungeon, and we always had to recruit people ourselves. It was troublesome. He had two accounts, and I had one. The team required three people." |
| Social assistance in the game | "I can also take him. So when I have time, I tell him to talk to him. I let my friend ask him, and he said it's okay, so I don't have to recruit people." |
| Long-term participation in the game | "I've been playing this game for almost two years, quite a long time." |
| Comparison cognition of game types | "This is also a monster-fighting leveling game, similar to Dungeon Fighter, Honor of Kings, especially similar to Dungeon Fighter." |
| Convenience of mobile devices | "I usually play on my phone, I'm too lazy to turn on the computer." |
| Diverse entertainment activities | "I would download a chess game, and then play the guitar for other games." |
| Relaxed attitude towards the game | "When I started playing this game, I didn't find it particularly fun, nor did I find it boring. I just felt that playing games is a way to relax, just like shopping for entertainment. So I'm not particularly obsessed." |
| Calm attitude towards the game | "I can play or not play, that's the feeling." |
| Repetitiveness cognition of the game content | "The main thing is that this game has been around for too long, and players feel that there's nothing special about it. It's too repetitive. The game itself is very repetitive." |
| Barrier-free design of the game | "It relies entirely on sound. All the monsters and things inside are based on sound. Just like sighted people read words, right? We just listen to its voice prompts." |
| Adjustment of game operation speed | "When you start playing, slow down the speech speed a bit, so you can hear more clearly. Once you get used to it, you can speed it up." |
| Impact of game operation speed on game efficiency | "Will slowing down or speeding up affect my monster-fighting speed, or the speed of fighting against others? Others won't be affected when they take you, but you will definitely be affected when you operate yourself." |
| New friends brought by the game | "The game has introduced me to quite a few friends." |
| No significant psychological impact of the game | "There hasn't been much of a psychological change." |
| Games as entertainment and social tools | "I didn't feel particularly happy or unhappy, I just felt it was okay. It's just a way to pass the time, and socializing is a very important aspect." |
| Construction of WeChat groups in the game community | "Yes, there are many, many groups. I'm in several guild groups." |
| Choosing and changing game guilds | "I initially joined a guild, but I felt it was too weak, so I left the guild. I didn't leave the group after leaving the guild, and then I joined another guild and entered another group." |
| Status and identity in the guild | "I'm in two guild groups, but I'm actually in one guild." |
| Views on conflicts in the game | "Ah, no, no. I won't get hit." |

## Table S7. ChatGPT (4-Turbo) open coding results for blind person 5

| **Code** | **Text** |
| --- | --- |
| Size and rank of the game guild | "The guild I'm in now is an A-level guild, and we always rank second in guild battles. There are about 100 people in it." |
| Perception of multi-account players | "He has several accounts by himself, I only have one. Some of them have several accounts, like Liu Siming has two. No, he plays with both accounts at the same time, both in the third area." |
| Perception of game complexity | "This game is actually a bit simple and straightforward." |
| Knowledge and comparison of other games | "Currently, I know there are several games we can play. One is 'Three Kingdoms Generals', one is 'Hongmeng Book of Heaven', one is 'Wind and Cloud Rise Again', and one is 'Listen to the World of Rivers and Lakes'." |
| Unique interaction mode in the game | "Because it has some interactions in the game. While playing, there's a feature where you can blow into the microphone, like when you want to hit harder, you can blow during a battle. This will increase its attack power." |
| Voice interaction in the game | "In this game, you can fight and talk. These are possible in the game." |
| Subjectivity of game attractiveness | "Actually, it may vary from person to person. I find it interesting, but others might not find it that way. It's subjective." |
| Control of game addiction level | "I might find it interesting when I first start playing, but I'm not that addicted. Some might be (addicted), but not necessarily." |
| Not staying up late for games | "Would you stay up late to play this game? Stay up late to play? Blind Person 5: No." |
| Impact of the game on daily life | "Although you play the game, to be honest, its attraction isn't that great. If there are other things to do, you'd do them. Mainly when you have nothing to do, it's just to pass the time." |
| Game as a way to pass the time | "There's nothing else. Maybe the other purpose is to change the way of entertainment. Don't always just listen to novels, maybe it's a change of entertainment." |
| Connection between game and socializing | "You will also want to meet some friends, right." |
| Neutral attitude of family towards the game | "Neither supportive nor opposed. If you have nothing to do, they won't say anything." |
| Connection between the game and the sense of social integration | "We will also have." |
| Joy brought by the game | "Yes, we will have. It's because sometimes when you fight in the game, it's quite interesting." |
| Influence of device on game type selection | "I used to play other games, but now I play fewer other games, mainly this game. I used to play other games, like poker, gold flower. Because now the iPhone doesn't support some things, if you use an Android phone, you can play more games." |
| Proportion of game players in the blind community | "Yes, I think maybe about one-third might play games." |
| Social interaction in the game | "I have a friend, who was also our colleague before. He would play games with me, and we would play games like this." |
| Stimulation and satisfaction brought by the game | "Sometimes it's quite stimulating and satisfying, it's quite stimulating and satisfying." |
| Habit of playing games during work breaks | "Yes, hmm." |
| Source of game information | "This game was introduced by my colleague just now, but after he introduced it to me, he didn't play it anymore. He played less, and later he didn't play much." |
| Difficulties and adaptations in the game learning process | "There will be some, yes, but after exploring for a while, it's easy to get started. Just explore by yourself for a while." |
| Real-life contact with teammates in the game | "There will also be, chat normally. Generally, if we have good teammates in the game, we will add WeChat, and we will chat about life, not just about the game, but also about other things." |
| Game as a way of socializing | "That's the way it is." |
| Actively seeking help when encountering game problems | "Will seek help." |
| Getting help through official answers | "There is usually a game guide in the game, and you can look at it if you encounter some problems." |
| Getting help from other players in the game | "Or if you can't, ask other people in the game." |
| Exploration and exploration in the early stage of the game | "When you first enter this game, you usually explore." |
| Adding friends in the early stage of the game | "You can usually add some friends." |
| In-game guidance | "At the beginning, there are always prompts, which are relatively simple. As long as you follow its instructions and operate step by step, there is no problem." |
| Using game guides to solve problems | "There is a game guide in the game itself, and you can look at it if you encounter some problems." |
| More difficulties in mobile phone operation than game operation | "We seek help, more in terms of mobile phone operation, which is relatively more difficult." |
| Problems with mobile software downloads | "Mobile phone operation, what system problems, or how to download mobile software, where to download these problems, there will be more of these." |
| Comparison of inconvenient game operations in the early days | "In the past games, like when people told you something, just like when we now call mobile phones, you have to find which room, which player, you can only do this. You have to query, please press 10, this thing is really not convenient." |
| Changes in games brought about by social media | "But with social media, there are many of these mutual aid games, which can be played directly." |
| Phenomenon of marriage in the game | "In our game, some people know each other and there is this kind of marriage between two people. There will be marriages in real life now." |
| Friends met through games are far away | "I might not have them in my daily life. Because I met them through games, they are from all over the place, generally far from here." |
| Establishment of game groups | "Our game is divided into factions. There's usually a faction leader who creates a group and pulls everyone in." |
| Game faction system | "Factions are based on your own wishes. You join whichever faction you want. There are many factions set up in the game. Or you can create your own faction in the game." |
| Competition and cooperation among factions | "Then you play the game. And there's competition between groups. Each faction will also create a group chat." |
| Regional factions in the game | "There are also factions like the Guizhou faction. There are indeed regional factions, like the Guizhou faction is for players from Guizhou." |
| Resources and money needed to establish a faction | "Yes, because establishing a faction also requires spending a lot of money and resources. Generally, ordinary people establishing a faction doesn't make much sense. It's more for those with ambition and funds." |
| Money needed to establish a faction | "It costs money to establish a faction, what's the use of that?" |
| Faction strength related to number of members | "If you spend money, you can have higher combat power, you can beat others, the number of people in your faction will increase, and the more people, the easier it is to beat others." |
| Spending in the game | "You joined a faction, you didn't create your own faction, right? Do you spend money in the game? I mean, do you make in-game purchases?" |
| Purchase of monthly cards in the game | "Yes, mainly what I spend on, like I've spent over 1000 yuan on this game, mainly buying things like monthly cards." |
| Trading of items in the game | "With so much investment, do you buy equipment?" |
| Offline trading of in-game items | "So you trade on WeChat, right?" |
| Price advantage of offline trading | "Is it cheaper to buy offline?" |
| Existence of online and offline trading in the game | "Both exist, there are in-game and out-of-game trades." |
| Posting trade information in the game group | "How do others know you're selling something? Do you post announcements?" |
| Imagining the game scene in the mind | "When you play the game, do you imagine the entire game scene in your mind? Do you build the entire map in your mind?" |
| Locating phone and game operations in the mind | "That is, locating things on the phone in the mind, and also remembering the entire map in the game. You remember everything in the game." |
| Posting information in the game | "You just said you buy things in the game, if you want to sell something, you can post it in your faction group, or you can post it in the game, right?" |
| Aggregative nature of the game: making friends and trading | "It's like the game also has a very aggregative nature, on the one hand, making friends, on the other hand, I can also carry out such material trades." |
| Playability of games for the visually impaired | "Like general games, there are relatively few games that blind people can play." |
| Sharing of games between the visually impaired and non-visually impaired | "Like other sighted people can play games, I can also play this game." |
| Feeling of difference between the visually impaired and non-visually impaired | "I feel that the gap between me and sighted people is not too big." |
| Inclusiveness of games for the visually impaired and non-visually impaired | "There are also some games that both sighted people and people with poor vision can play." |
| Influence of games on social integration of the visually impaired | "It can be more integrated." |
| Perception of the psychological impact of games on the visually impaired | "There will be some feelings." |
| Perception of the game experience of others by the visually impaired | "I think this might be possible." |
| Popularity of games among the visually impaired | "Most people still like it quite a bit." |
| Role of games in the lives of the visually impaired | "Now it's just to add some fun to daily life." |
| Joy of life brought by games | "Yes, this sense of fun." |
| Degree of influence of games on the lives of the visually impaired | "Yes, there are changes, there is help, it shouldn't be said to be great, but it really can help." |
| Influence of games on the social life of the visually impaired | "In terms of daily social interactions and enriching leisure time, the help should be quite significant." |
| Size of the game faction | "There are over 100 people in the faction I'm in." |
| Organization of the game faction | "Our faction and other factions are like this, not just people from our own faction." |
| Individual and collective combat power in the game | "Individual combat power and collective combat power." |
| Game's demand for individual strength and compensation for collective advantages | "If you have high individual combat power, maybe 1 person can fight 10 people." "Maybe there are more people, more people can go online, and play in this way together." |
| Cross-regional connections brought by the game | "The people inside are from all over the place." |
| Influence of games on friendship building among the visually impaired | "There will also be some that turn into relatively good friends." |

## Table S8. ChatGPT (4-Turbo) open coding results for blind person 6

| **Code** | **Text** |
| --- | --- |
| Games as a tool to pass time | "Not much has changed. Now we're just bored, chatting and playing games. It feels like killing time." |
| Game interest over social motivation | "Making friends might be a possibility, but if I meet someone good and can chat well, I might make friends, but I'm not there just to make friends." |
| Games as a way to alleviate loneliness | "Yes, when I'm lonely and bored with no one to talk to, I might play. I just want to play for a bit." |
| Game players' investment vs. rewards | "I'm just an ordinary player and I haven't spent much money in the game. I've played this game for over a year, but I don't think I've played for a long time. Some have played for two or three years." |
| Impact of games on emotions and psychology | "I think games have helped me emotionally and psychologically. When I'm bored or upset, playing games makes me feel better and not always think about unhappy things. It at least diverts my attention." |
| Joy from games and lack of self-worth | "Yes, playing games makes me happy, but it hasn't changed my perception and attitude towards myself. I don't feel an increase in self-worth because of playing games." |
| Subjectivity of game experience | "For me, there's nothing wrong. I haven't spent much money in the game, so I haven't lost anything." |
| Social interaction within games | "I mainly play with acquaintances. Most of these acquaintances are people I met in the game, forming a fixed group of players." |
| Connection between game friendships and real life | "They listen to me when I'm down and provide psychological support, but not much else." |
| Online game community communication and non-relevance to location | "Yes. Now, distance is no longer an issue. Online communication is very convenient, like chatting. If you want to meet, you can do it tomorrow morning. Just take the high-speed train, it's up to you if you want to." |
| Openness and privacy of the game community | "No, if we've agreed in advance, we'll choose private chat. In group chats, whoever is free can join. This mechanism is very open, everyone can talk to each other. If we don't want others to join, we'll choose private chat, no need to do it in the group." |
| Minimal impact of games on blind person's life and attitude | "Nothing has changed, I think everyone has their own way of life." |
| Positive impact of games on others' emotional lives | "Some have found partners in games." |
| Negative impact of games on others' emotional lives | "Some have had their families broken up because of games." |
| Awareness of issues games might bring in long-distance relationships | "Especially for those in long-distance relationships, this problem is more likely to arise." |
| Impact of games and other social platforms on family relationships | "Many people have had their families broken up because of chatting on WeChat or TikTok, and many have found partners in games." |
| Long-distance relationships in games | "I have many classmates who became couples because of playing games. For instance, one might be in Beijing and the other in Harbin. Despite the geographical distance, they get along quite well." |
| Relationship between game fun and friends' support | "Games indeed bring some fun, but I don't think I get more support from friends because of it." |
| Games' lack of contribution to life fulfillment | "As for whether life becomes more fulfilling because of it, I feel it still lacks a bit." |
| Games as a tool to pass time | "I keep playing it mainly because it helps me kill time when I'm bored." |
| Social gains in games: meeting new friends | "In the game, I indeed made two good friends." |
| Impact of games on daily life: mainly social and psychological | "Yes, I did gain something socially and psychologically from the game. But I don't see it as a big deal." |
| Secondary role of games in daily life | "Most of my time and energy are spent on taking care of my child. I only play games when my child is at school and I have some free time." |
| Role of games in alleviating loneliness | "Everyone feels lonely sometimes, including me. But I find that playing games can alleviate my loneliness to some extent." |
| Game style: not pursuing advanced, mainly casual play | "Most of the time, I'm fighting regular monsters, not pursuing the advanced ones." |
| Attitude towards games in the player community: mainly for killing time and socializing | "He said he also uses games to pass the time, and playing hasn't brought significant changes to his life. He feels there might be some improvement in the social aspect since he made many friends through the game." |
| Phenomenon of games as social tools: expanding social circles, finding partners | "Some people do use it that way. I've heard of some who play games to expand their social circles, even to find girlfriends." |

## Table S9. ChatGPT (4-Turbo) open coding results for blind person 7

| **Code** | **Text** |
| --- | --- |
| Interviewee's age | "Yes, I'm 18 now." |
| Interviewee's approximate age | "Just got my own phone." (Phones are typically obtained around the age of 13) |
| Origin of the interviewee's vision problem | "It's congenital." |
| Time interviewee started playing the game | "I started playing this game in October 2018." |
| Situation when the interviewee started playing | "At that time, I just got my own phone but didn't know what to do with it." |
| Game player's frequency of playing | "I used to play quite frequently, but recently I occasionally log in to check and participate in guild activities." |
| Adjustment in game player's frequency | "I log in about two or three times a week." |
| Reason for adjustment in game frequency | "I'm in school now, and there's a rule that we can't use phones at night." |
| Game player's duration of play | "Now when I log in, I usually play for about half an hour, mainly to check on the guild and communicate with other players." |
| Reason for starting the game | "Then I saw someone playing this game in a special group, so I downloaded it to try. I found it to be blind-friendly, with a storyline and battles, so I started playing." |
| Game's appeal: storyline and battles | "I got addicted as soon as I started playing, finding the storyline very engaging." |
| Novelty of the game | "Yes, it felt like opening a new world." |
| Game's accessibility design: map design, sound effects, and storyline | "What I like most is its accessibility design, which allows me to fully immerse myself in the game. For example, the accessible map is very well done. I also love its sound effects and storyline; the sound effects are dynamic and excellent, and the storyline is coherent." |
| Game's social function: established friendships | "Yes, I joined a guild in this game, and through the guild, I made many friends. We also communicate a lot on WeChat, so I think the game does well in terms of social interaction." |
| Game's social function: WeChat group communication | "We also have a lot of communication on WeChat." |
| Initial intention and subsequent change in the game | "Yes, I met quite a few people, which might be the most attractive part for me. Initially, I just played because I found the game interesting, playing alone without thinking about anything else." |
| Game's social function: Changes in social behavior | "Initially, I played with strangers. Gradually, I started playing with acquaintances. Later, I found some regular teammates in the game group. We'd play games and participate in activities at fixed times. Slowly, strangers became acquaintances." |
| Help from game friends | "They've helped me a lot. For instance, when I'm feeling down or facing difficulties, they'd assist and counsel me. When I'm bored, I'd chat with them." |
| Emotional support from game friends | "Mainly emotional issues or minor life troubles. I can chat with them, and they'd offer advice and comfort." |
| Technical support from game friends | "Yes, they help me both in-game and in real life. For example, if I have problems using the phone's screen-reading function, I'd ask them, and they'd guide me. Or if I'm stuck in the game, they'd advise me on how to proceed." |
| Possibility of game friends becoming offline friends | "I haven't met the friends I made in the game. We're all students, so meeting hasn't been discussed. However, we have a good relationship on WeChat." |
| Diversified social interactions in games: Choosing friends | "I've encountered various types of people, but I can choose whom I want to befriend." |
| Establishing social relationships in games: Connecting through commonalities | "It feels like finding a common point with game friends, and then establishing a connection." |
| Equality in games: Not limited by age or status | "Yes, age isn't an issue. We're all equal, and no one judges the other." |
| Game's ease of use: Clear operation prompts | "You can swipe up to skip the storyline, and then the game tells me what to do next. I just follow the prompts." |
| Game's convenience: Auto-pathfinding feature | "You can also auto-pathfind." |
| Game's challenges: Level restrictions | "This restriction does exist, but there are exceptions." |
| Assistance in games: High and low-level players helping each other | "For instance, if you're at a lower level, you can challenge high-level dungeons with higher-level players. And even if you're at a high level, you might not necessarily get good equipment, so high-level players would also assist lower-level players." |
| Patience cultivated by games: Controlling emotions | "It's a process that requires patience. I'd stand there, sometimes facing opponents close to my level, and lose for various reasons, which would make me angry. But if I lose often or over a long period, I'd become more patient. So, this game also helps me maintain patience in life." |
| Exploratory nature of games: Discovering and choosing strategies | "No matter what game you play, it requires an exploratory spirit. You have to discover which method works best. For instance, just investing money might not help you level up, but if you change your approach, you might find leveling up faster. These methods need self-exploration." |
| Joyful experience in games: Satisfaction and self-identification | "I feel games are a form of self-satisfaction. Even though I know it's virtual, I feel like I am that character. I strongly identify with the in-game character." |
| Multi-faceted emotional impact of games: From satisfaction to dissatisfaction | "Yes, this sense of achievement makes me happy. I feel satisfied and content with my achievements. But there are also negative emotions, like tension, fatigue, or restlessness. When facing opponents of similar strength, I might feel anger and complaints. For instance, if I predict a loss and then lose, I'd be upset. But I won't feel tired; I'd continue playing. Also, if I feel unlucky, I might complain about my bad luck." |
| Difficulties in using smartphones: Challenges in finding information | "I might feel frustrated if I can't find the information I need or the app I want. For instance, there are few apps suitable for the blind, so sometimes I can't find resources I need, like video resources." |
| Difficulties in using smartphones: Compatibility and unclear functions | "Some apps, like iQiyi, might not support my screen-reading software, or they run very laggy, and I can't even find the play button." |
| Expectations for games and phones: Development of apps for the blind | "For phones, I think apps suitable for the blind can be developed. Many screen-reading software options are paid, and the free ones have many issues." |
| Suggestions for games and phones: Enhancing clarity of functions | "Some button functions are unclear, leaving me puzzled." |

## Table S10. ChatGPT (4-Turbo) open coding results for blind person 8

| **Code** | **Text** |
| --- | --- |
| Interviewee's age: 14 years old | "You're 14 this year, right?" "Yes." |
| Interviewee's vision: Congenital blindness | "What's your current vision status? Congenital or acquired?" "It's congenital." |
| Gaming experience: Played for 1-2 years | "When did you start playing this game? How long have you been playing?" "I think it's been about one to two years." |
| Gaming frequency: From school start to mid-term | "What's your current gaming frequency? How often do you play in a week?" "I usually play from the start of the school term until mid-term, then I don't log in much." |
| Game content preference: Storyline | "Initially, I played the game mainly for the storyline. I wasn't interested in fighting various things in the game, just liked listening to the story." |
| Game's attraction: Storyline | "I like watching those storylines." "You like the storyline because it's attractive, right?" |
| Motivation to choose the game: Curiosity | "Mainly out of curiosity, interested in something new." |
| Game's social aspect: Minimal, only with classmates | "Do you socialize in the game?" "Not really, just play with classmates." |
| Social behavior content: Game tactics and storyline discussion | "Because I level up slowly, I ask them for leveling tactics, and then discuss some storylines." |
| Game's social aspect: Looking for dungeon partners in the guild | "Maybe sometimes? Like when I want to do dungeons, I might look for some people from the guild." |
| Game partner choice: Mainly known classmates | "Who are your regular game partners? Is it always random? You have two groups of game partners, one is classmates you already know, and the other is from the guild or randomly matched, right? Which is more?" "Playing with classmates is more frequent." |
| Social environment: Same dormitory or class classmates | "Mostly from the same dormitory or the same class." |
| Game's impact on social relationships: Deepened relationship with classmates | "So you were already good friends, and then you discussed and played this game together, right?" "Yes." |
| Initial motivation for the game: Seeking a fresh experience due to boring studies | "What was your initial motivation to try this game? Why did you want to play it? You said because studying was boring, you wanted to try something fresh out of curiosity, right? Is my understanding correct? Any other reasons, like the storyline or other attractive aspects?" "Yes, I felt studying was boring and wanted to try something fresh. I was looking for a game suitable for me." |
| Game experience: Suitable initially, slow leveling later | "Do you think this game suits you?" "I felt it was suitable in the beginning, but leveling up felt too slow later on." |
| Game experience: Slow updates | "Updates were once a week later on, which felt too slow." |
| Game's impact on devices: Long hours drained battery, affecting phone usage | "I had the game running on my previous phone, and the battery got ruined." "Yes, I had the game running almost all day for those few days." |
| Gaming behavior: Long hours of tasks | "Why did you keep it running? For leveling up? Does the game level up automatically when you keep it running?" "No, there are some tasks in the game, like hanging out in the mountain spring, which takes over an hour each time." |
| Game's impact on social relationships: Strengthened relationships with acquaintances, no significant expansion of social circle | "Do you think playing the game has helped your social relationships? Like getting closer to close friends and expanding your social circle, right?" "Maybe I've gotten closer to acquaintances, but I wouldn't say it expanded my social circle." |
| Social content in the game: Discussing life, game discussions | "What do you usually discuss with people you meet in the game?" "Sometimes when teaming up, they chat, and I might join in. I remember once meeting an older graduate from our school and discussing life after graduation with him." |
| Game's emotional impact: A way to vent negative emotions | "So, you play games when you're upset, is it a way to vent emotions?" "Yes, playing games makes me happy, and then I forget about the things that upset me." |
| Gaming habits: After class, after self-study | "Any other gaming habits or timings?" "Usually after class or after self-study, I don't play much on weekends." |
| Game's impact on interests: Curiosity about the storyline | "On one hand, this game consumes less battery, and on the other, I'm genuinely curious about the game's storyline. So, I level up as quickly as possible. Every time a bit of the story is unlocked, I listen to it carefully." |
| Game's help: Emotional support, minor practical problem-solving | "So, you can discuss with people you meet in the game and they comfort you. This is emotional support. Do they help in other ways? Like if you're stuck in the game, or in studies, or in using electronic products. Do you seek information and support?" "I generally like to figure things out. If I encounter a problem, I try to solve it myself. After all, I just have low vision, so I don't usually need help with most issues." |
| Sense of achievement from self-breakthrough | "It's a mix of feelings. Sometimes, when I surpass my highest level from the previous day, I feel a great sense of achievement." |
| Anger from competitive failure | "Sometimes, when I'm competing with someone and I'm just about to win, I feel very angry." |
| Short gaming time leads to competitive disadvantage | "Compared to my classmates, I might have played this game for a shorter time. Even if we started on the same day, their levels would be higher than mine." |
| Self-evaluation drops due to game ranking | "I didn't play much later on. Basically, in our circle, I'm usually the last one." |
| Small-scale campus game community | "Our school has few students to begin with, just about ten or so. We communicate privately." |
| Borrowing others' game accounts to experience guild interactions | "But I once borrowed a high-level account from a classmate, and that account was part of a guild." |
| Game performance issues: Slide lag | "I feel the recent game is a bit laggy. I used to slide very fast, but now the system, for some reason, can't keep up. I've already slid to the next step, but it's still on the previous one. Sometimes it just freezes." |
| Games alleviate boredom | "To alleviate boredom. Otherwise, during boring times, I'd just be bored with nothing to alleviate it." |
| Games alleviate loneliness | "It does help a bit. For instance, when I play in the social arena and win, it helps alleviate feelings of loneliness." |
| Tension in games: Tense when health is low | "When competing with others, if I see my health is running low, and I'm just a few points away from losing, I feel very tense." |
| Pressure from games: Level-up pressure | "Sometimes, I feel I haven't reached a new level, but I know I should have. That brings a kind of pressure." |
| New insights from games | "Yes, I find this game interesting because it gives me new insights and deepens my understanding of certain principles." |
| Games enhance cognitive abilities | "Yes, and it might even improve my cognitive abilities to some extent because it involves a lot of elements. Sometimes, you even need to calculate. I think that's great." |
| Participation in the game's first recharge event | "Usually, it's the first recharge, which is a special offer they have for every new account. They give more during this event." |
| Participation in special game events | "Yes, or some super-value events. I'd participate in those." |
| Multiple account registration status | "I basically have an account in every region, and in some regions, I have two. Yes, you can register two accounts with WeChat and a phone number. I have two phone numbers." |
| Game recharge amount status | "The first recharge is only 6 yuan. Yes, I might have recharged 15 once because I was missing something and needed it urgently. Yes, if I could recharge up to 100, that would be good. That should be the total amount." |

# ChatGPT (4-Turbo) axial coding results

## Table S11. ChatGPT (4-Turbo) axial coding results for blind person 1-2

| **Theme** | **Sub-theme** | **Text** |
| --- | --- | --- |
| game motivation | Subtopic 1: Killing time | Blind Man 1: Games are seen as time killers |
|  |  | Blind Man 2: Initial motivation for playing the game: killing time |
|  | Subtheme 2: Social needs | Blind Man 1: Games as social tools |
|  |  | Blind Man 2: Initial motivation for playing the game: socializing; social function of the game: making friends; reason for staying in the game: social relationships |
|  |  | Blind Man 1: Games as social tools |
|  | Subtheme 3: Pursuing a sense of achievement/vanity | Blind Man 1: Opinions on Game Achievement |
|  |  | Blind Man 2: The source of the sense of accomplishment in the game: Becoming the number one in the martial arts world; The source of the sense of accomplishment in the game: Satisfying vanity |
| Game experience | Subtheme 1: Game activity preferences | Blind Man 1: Favorite game sessions: imperial examinations and answering questions while drinking |
|  | Subtheme 2: Game Characters and Identity | Blind Man 2: Game Identity: Gang Leader |
|  | Subtheme 3: Game performance and status | Blind Man 2: Changes in the game ranking: from strong to weak ; dissatisfaction with the state of the game: because no more recharges, it has become less powerful |
|  | Subtheme 4: How to first contact the game | Blind Man 1: Initial contact with the game: introduction by friends |
|  |  | Blind Man 2: How to get in touch with the game: Introduction by classmates |
|  | Subtheme 5: The appeal of games | Blind Man 2: The Attraction of the Game: Realizing the Fantasy Dream |
| Game consumption and recharge attitude | Subtheme 1: Consumption attitude | Blind man 1: Attitude towards game consumption: No payment |
|  |  | Blind Man 2: Recharge amount: hundreds of thousands; pressure to recharge: in order not to hold friends back |
|  | Subtopic 2: Reflection on recharging | Blind Man 1: Opinions on in-game recharge |
|  |  | Reflection after the recharge behavior: waste; change of attitude towards game investment: from recharge to regret |
| The impact of gaming | Subtheme 1: Negative effects | Blind Man 2: Negative review of the game: Devil; Negative effect of the game: Spending too much; Warning of addictive game: Devil, addictive |
|  | Subtheme 2: Positive impact | Blind Man 1: Gains from Games: Diversified Entertainment Methods |
|  |  | Blind Man 2: Positive takeaways from play: Broad thinking; Positive takeaways from play: Judgment of character |
|  | Subtheme 3: Negative emotions caused by games | Blind Man 2: Negative Emotions Caused by Games: Sadness of Failure |
| Game awareness and attitudes | Subtopic 1: The difference between games and reality | Blind Man 2: Cognition of virtuality in games: the difference between games and reality |
|  | Subtheme 2: Attitude towards games | Blind Man 1: The original intention of the game: self-entertainment, not being controlled by the game; the intermittent attitude towards the game |
|  |  | Blind Man 2: Humanity Issues in Games: Revenge; Attitude towards the Virtual World: There is no need to pay too much attention to virtual things |
|  | Subtheme 3: Opinions and evaluations on the game | Blind Man 1: Opinions on In-Game Recharge |
|  |  | The Blind Side 2: Negative Reviews of the Game: The Devil |
|  |  | Blind Man 2: Game Addiction Warning: Devil, Addictive |
| Game improvement suggestions |  | Blind Man 1: Suggestions for game improvement: add mini-games |

## Table S12. ChatGPT (4-Turbo) axial coding results for blind person 3-4

| **Theme** | **Sub-theme** | **Text** |
| --- | --- | --- |
| Game experience and attitude | Subtheme 1: Game contact history and current situation | The Blind Side 3: Game Contact History |
|  |  | Blind Man 3: It’s officially time to start the game |
|  |  | Blind Man 3: Time to Interrupt Gameplay |
|  |  | Blind 4: Enter the game via social networks |
|  |  | Blind Man 4: Being in the Game for the Long Term |
|  | Subtheme 2: Attitude towards games | Blind Man 3: Reason why the game has been temporarily abandoned |
|  |  | The Blind Side 3: Reasons for the Game's Revival |
|  |  | Blind Man 3: Boredom caused by excessive gaming |
|  |  | Blind Man 4: A relaxed approach to gaming |
|  |  | Blind Man 4: Calm attitude toward games |
|  | Subtheme 3: Time consumption and efficiency in games | Blind Man 3: Daily Game Hours |
|  |  | Blind Man 4: Adjustment of the operation speed in the game |
|  |  | Blind Man 4: The impact of game operation speed on game efficiency |
| Social Motivation and Value of Games | Subtheme 1: Social Motivation of Games | Blind 3: The Social Motivation of Games |
|  |  | Blind Man 4: Teamwork in the Game |
|  |  | Blind Man 4: Social Mutual Aid in Games |
|  | Subtheme 2: Social value of games | Blind Man 3: The Social Value of Games |
|  |  | Blind Man 3: Social Value in Games |
|  |  | Blind Man 4: New Friends the Game Brings |
|  |  | Blind Man 4: Games as recreation and social tool |
| In-game investments and attitudes | Subtheme 1: In-game investments | Blind Man 3: Total In-Game Investment |
|  | Subtheme 2: Attitude towards in-game recharge | Blind Man 3: Attitude towards in-game recharge |
| Social interactions and experiences in games | Subtheme 1: Main social objects and interactions in the game | Blind 3: The main social object of the game |
|  |  | Blind Man 3: Observations and Attitudes towards Other Players |
|  |  | Blind Man 4: WeChat Group Construction of Game Community |
|  |  | Blind 4: Choose and Change Game Gangs |
|  |  | Blind Man 4: Position and Identity in the Gang |
|  | Subtheme 2: Perceptions and handling of in-game conflicts | Blind Man 3: Encountered Unprovoked Attack |
|  |  | Blind Man 3: Encountered Continuous Attack |
|  |  | Blind Man 3: Pressure from Powerful Players |
|  |  | Blind Man 4: A Perspective on In-Game Conflict |
| Games bring experience and impact | Subtheme 1: Emotions and experiences brought about by games | Blind Man 3: Negativity brought about by gaming |
|  |  | Blind Man 3: Brief Happiness in the Game |
|  |  | Blind Man 4: Games have no obvious impact on mental state |
|  | Subtheme 2: The impact of games on life and work | Blind Man 3: The Impact of Games on Life Fulfillment |
|  |  | Blind Man 3: The Impact of Gaming on Work |
|  |  | Blind 3: Game social activities extend to real life |
|  |  | Blind Man 3: Improving life through social networking through games |
|  |  | Blind Man 3: Games consume time |

## Table S13. ChatGPT (4-Turbo) axial coding results for blind person 5-6

| **Theme** | **Sub-theme** | **Text** |
| --- | --- | --- |
| Social Motivation of Games | Subtheme 1: Players’ social interactions | The game’s unique interactive approach |
|  |  | In-game voice interaction |
|  |  | The connection between games and social media |
|  |  | Social interaction in games |
|  |  | Social interaction within the game |
|  | Subtheme 2: Relationships built in the game | Real life contact between teammates in the game |
|  |  | Most of the friends I met through games are far away |
|  |  | The connection between gaming friendships and real life |
|  | Subtheme 3: Games as social tools | Games as a way to socialize |
|  |  | Social gains in the game: meet new friends |
|  |  | The phenomenon of games as social tools: broaden your social circle and find a partner |
|  | Subtheme 4: Gaming Community | Game gang size and level |
|  |  | Create a group in the game |
|  |  | Gang system in the game |
|  |  | Game gang size |
|  |  | How game gangs are organized |
|  |  | Communication styles and location independence in online gaming communities |
|  |  | The openness and privacy of gaming communities |
| The impact of gaming | Subtheme 1: Impact on daily life | The impact of games on daily life |
|  |  | The impact of games on Blind Man 6’s personal life status and attitude is minimal. |
|  |  | The impact of games on daily life: mainly focused on social and psychological aspects |
|  | Subtheme 2: Impact on psychological state and emotions | The subjectivity of game appeal |
|  |  | The joy brought by games |
|  |  | The impact of games on personal emotions and mental states |
|  |  | The excitement and satisfaction brought by games |
|  |  | games on visually impaired people |
|  |  | Games as a way to relieve loneliness |
|  |  | The impact of games on personal emotions and mental states |
|  |  | The joy brought by gaming and the lack of self-worth |
|  |  | The role of games in alleviating loneliness |
| Game operation and learning | Subtheme 1: Game Mastery and Learning | Game information source |
|  |  | Difficulties and adaptations in the game learning process |
|  |  | Use game guides to solve problems |
|  | Subtheme 2: Challenges of game operation | Mobile phone operation is more difficult than game operation |
|  |  | Problems with downloading mobile software |
|  |  | Inconvenient operation compared to earlier games |
| In-game transactions and spending |  | spend in game |
|  |  | Purchase an in-game monthly pass |
|  |  | In-game item trading |
|  |  | Offline trading of in-game items |
|  |  | Price advantage of offline transactions of in-game items |
|  |  | The existence of in-game online and offline transactions |
|  |  | Post transaction information in the game group |
| Game experience and cognition | Subtheme 1: Game experience | Perceptions of game complexity |
|  |  | The game’s unique interactive approach |
|  |  | In-game voice interaction |
|  |  | Difficulties and adaptations in the game learning process |
|  |  | Exploration and exploration in the early stages of the game |
|  |  | In-game instructions |
|  |  | Build a picture of the game in your mind |
|  |  | Position mobile phone and game operations in your mind |
|  |  | The personal subjectivity of gaming experience |
|  |  | Game mode: Do not pursue advanced gameplay, focus on ordinary gameplay |
|  | Subtheme 2: Game cognition | Awareness of multi-account players |
|  |  | Awareness and comparison of other games |
|  |  | Proportion of gamers in the blind community |
|  |  | Game information source |
|  |  | The shareability of games between visually impaired and non-visually impaired people |
|  |  | Popularity of games among visually impaired people |
| The impact of gaming on interpersonal and family relationships |  | Real life contact between teammates in the game |
|  |  | friends met through games |
|  |  | Marriage in the game |
|  |  | The situation of friends who got married through games |
|  |  | of marriage through games among visually impaired people |
|  |  | The impact of games on interpersonal relationships |
|  |  | The difference between friends made through games and friends in real life |
|  |  | Marriage through games |
|  |  | Contact with friends you met in the game in real life |
|  |  | The real-life impact of friends you meet through gaming |
|  |  | The prevalence of marriage through games among visually impaired people |
|  |  | Cases of getting married through games |
|  |  | The impact of gaming on family relationships |

## Table S14. ChatGPT (4-Turbo) axial coding results for blind person 7-8

| **Theme** | **Sub-theme** | **Text** |
| --- | --- | --- |
| Personal information and background | Sub-theme 1: Age range of respondents | Respondent age |
|  |  | Respondent age (approximate) |
|  |  | Respondent’s age: 14 years old |
|  | Subtheme 2: Respondents’ origins of vision problems | Origin of Respondent’s Vision Problem |
|  |  | Respondent’s vision status: congenitally blind |
| Gaming behaviors and habits | Subtopic 1: Game start time and environment | When the respondent started playing the game |
|  |  | The context in which the respondent started playing the game |
|  |  | Gaming experience: Played for 1-2 years |
|  | Subtheme 2: Frequency and duration of games | How often gamers play |
|  |  | Game frequency adjustment for gamers |
|  |  | Gamers’ gaming time |
|  |  | Game frequency: every semester |
|  | Subtopic 3: Changes in game frequency and reasons | Reasons why gamers adjust their gaming frequency |
|  | Subtheme 4: Drivers to start playing games | Reasons to start playing games |
|  |  | Motives for choosing early in the game: Learning is boring, seeking fresh experience |
| game motivation | Subtheme 1 : Novelty and Curiosity (Entertainment and Recreation) | Reasons to start playing games |
|  |  | The novelty of the game |
|  |  | What drives game selection: Curiosity |
|  |  | Motives for choosing early in the game: Learning is boring, seeking fresh experience |
|  | Subtheme 2 : Game content and structure (plot and combat, challenge and difficulty, operation and design) | The appeal of the game: plot and combat |
|  |  | Accessibility design of the game: map design, sound effects and plot settings |
|  |  | The challenge of the game: level restrictions exist |
|  |  | The game is easy to use: clear operation prompts |
|  |  | Game content preference: plot |
|  |  | Source of game attraction: plot |
| Social interaction in games | Subtheme 1: Social methods in games | Game social function: WeChat group communication |
|  |  | Game socialization: basically not played, only played with classmates |
|  |  | Game socialization: looking for dungeon partners in the guild |
|  | Subtheme 2: The impact of games on social behavior | Social functions of games: changes in social behavior caused by games |
|  |  | The impact of games on social relationships: deepening relationships with classmates through games |
|  |  | The impact of games on social relationships: improving relationships with acquaintances without significantly expanding social circles |
|  | Subtheme 3: Possibility of friends in the game turning into friends in real life | Possibility of gaming friends becoming offline friends |
|  | Subtheme 4 : The establishment and development of social relationships in games | Building social relationships in games: connecting through commonalities |
|  |  | Social environment: classmates in the same dormitory or the same class |
|  | Subtheme 5: Selection of gaming partners | Social Diversity in Games: The Independent Choice of Making Friends |
|  |  | Game partner selection: mainly known classmates |
| The impact of games on personal emotions and feelings | Subtheme 1: Game appeal and novelty | The appeal of the game: plot and combat |
|  |  | The novelty of the game |
|  |  | Source of game attraction: plot |
|  |  | What drives game selection: Curiosity |
|  | Subtheme 2: Joyful experience in games | Joyful experiences in games: satisfaction and self-identity |
|  |  | The impact of games on interest: curiosity about the plot |
|  | Subtheme 3: The multifaceted impact of games on emotions | The multifaceted effects of gaming on mood: from satisfaction to dissatisfaction |
|  |  | The emotional impact of games: Resolving unhappy emotions |
|  | Subtheme 4: The impact of competitive failure and game ranking on emotions | The anger of losing in competition |
|  |  | Decline in self-evaluation caused by game rankings |
|  | Subtheme 5: Games relieve boredom and loneliness | Games relieve boredom |
|  |  | Games relieve loneliness |
| Game accessibility and design | Subtopic 1: The accessibility design of the game includes map design, sound effects and plot settings | Accessibility design of the game: map design, sound effects and plot settings |
|  | Subtheme 2: Ease of use and convenience of the game | The game is easy to use: clear operation prompts |
|  |  | Game convenience: automatic pathfinding function |
|  | Subtheme 3: Difficulty using smartphones | Difficulties in Smartphone Use: The Challenge of Finding Information |
|  |  | Difficulties in using smartphones: Compatibility and unclear functionality issues |
|  |  | Game performance issues: sliding lag |
|  | Subtheme 4: Expectations and suggestions for games and mobile phones | What to expect from games and mobile phones: App development for blind people |
|  |  | Recommendations for games and mobile phones: Improve the clarity of feature labels |
| Game experience and technical support | Subtheme 1: Game Challenges and Strategies | The challenge of the game: level restrictions exist |
|  |  | Explorability in games: strategy discovery and choice |
|  |  | Competitive disadvantages caused by short game time |
|  |  | Assistance of the game: high and low level players help each other |
|  | Subtopic 2: Technical Support | Technical support provided by gaming friends |
|  |  | Difficulties in using smartphones: Compatibility and unclear functionality issues |
|  |  | Game performance issues: sliding lag |
| The life impact of gaming | Subtheme 1: Impact on daily life | The device impact of the game: Playing for a long time consumes a lot of power and affects the use of mobile phones. |
|  |  | Games relieve boredom |
|  |  | Games relieve loneliness |
|  | Subtheme 2: Improvement of life skills | Patience cultivation brought by games: controlling emotions |
|  |  | Games improve thinking ability |
|  | Subtheme 3 : Social influence (the influence of social relationships, social environment, community behavior) | Possibility of gaming friends becoming offline friends |
|  |  | Game perception of the surrounding environment: the impact of sound effects |
|  |  | The impact of games on social relationships: deepening relationships with classmates through games |
| game economy |  | Participation in the game’s first recharge event |
|  |  | Participation in game special events |
|  |  | Multiple account registration status |
|  |  | Game recharge amount |

## Table S15. ChatGPT (4-Turbo) axial coding results for blind person 1-8

| **Theme** | **Sub-theme** |
| --- | --- |
| Game Motivation and Attitude | Subtheme 1: Game motivation (blind person 1-2, blind person 7-8) |
|  | Subtheme 2: Killing time (blind person 1-2) |
|  | Subtheme 3: Social needs (blind person 1-2, blind person 3-4, blind person 5-6) |
|  | Subtheme 4: Pursuing a sense of achievement/vanity (blind person 1-2) |
|  | Subtheme 5: History and current situation of game contact (blind people 3-4) |
|  | Subtheme 6: Time consumption and efficiency in games (Blind 3-4) |
|  | Subtheme 7: Novelty and Curiosity (Entertainment and Recreation) (Blind People 7-8) |
|  | Subtheme 8: Game content and structure (plot and combat, challenge and difficulty, operation and design) (Blind 7-8) |
| Game experience | Subtheme 1: Game activity preferences (blind people 1-2) |
|  | Subtheme 2: Game Role and Identity (Blind 1-2) |
|  | Subtheme 3: Game performance and status (Blind 1-2) |
|  | Subtheme 4: First contact with the game (blind people 1-2) |
|  | Subtheme 5: Attractiveness of games (blind people 1-2, blind people 7-8) |
|  | Subtheme 6: Main social objects and interactions in games (Blind 3-4) |
|  | Subtheme 7: Perceptions and handling of in-game conflicts (Blind 3-4) |
|  | Subtheme 8: Game experience (blind people 5-6) |
| Game consumption and investment | Subtheme 1: Game consumption and recharge attitude (blind people 1-2) |
|  | Subtheme 2: Consumption attitude (blind people 1-2) |
|  | Subtopic 3: Reflection on recharging (blind person 1-2) |
|  | Subtheme 4: In-game investments and attitudes (Blind 3-4) |
|  | Subtheme 5: In-game investments (Blind 3-4) |
|  | Subtheme 6: Attitude towards in-game recharge (Blind 3-4) |
|  | Subtopic 7: In-game transactions and consumption (blind people 5-6) |
| The impact of games on life | Subtheme 1: Impact of gaming (blind people 1-2, blind people 5-6) |
|  | Subtopic 2: Negative effects (blind people 1-2) |
|  | Subtheme 3: Positive Impact (Blind People 1-2) |
|  | Subtheme 4: Negative emotions caused by games (blind people 1-2) |
|  | Subtheme 5: The impact of games on life and work (blind people 3-4) |
|  | Subtheme 6: Impact on daily life (blind people 5-6) |
|  | Subtheme 7: Impact on mental state and emotions (blind people 5-6) |
|  | Subtheme 8: The impact of games on interpersonal and family relationships (Blind 5-6) |
|  | Subtheme 9: Impact on daily life (blind people 7-8) |
|  | Subtheme 10: Improvement of life skills (blind people 7-8) |
| Game operation and learning | Subtheme 1: Game Mastery and Learning (Blind People 5-6) |
|  | Subtheme 2: Challenges in game operation (blind people 5-6) |
| Social interaction in games | Subtheme 1: Player social interaction (Blind 5-6) |
|  | Subtheme 2: Relationships built in the game (Blind 5-6) |
|  | Subtheme 3: Games as social tools (blind people 5-6) |
|  | Subtheme 4: Gaming Community (Blind People 5-6) |
|  | Subtheme 5: Social methods in games (blind people 7-8) |
|  | Subtheme 6: The impact of games on social behavior (blind people 7-8) |
|  | Subtheme 7: The possibility of friends in games turning into friends in real life (blind people 7-8) |
|  | Subtheme 8: Establishment and development of social relationships in games (blind people 7-8) |
|  | Subtheme 9: Selection of game partners (blind people 7-8) |
| Game accessibility and design | Subtheme 1: The accessibility design of the game includes map design, sound effects and plot settings (blind people 7-8) |
|  | Subtheme 2: Ease of use and convenience of games (blind people 7-8) |
|  | Subtheme 3: Difficulty using smartphones (blind people 7-8) |
|  | Subtheme 4: Expectations and suggestions for games and mobile phones (blind people 7-8) |
| Personal information and background | Sub-theme 1: Age range of respondents |
|  | Subtheme 2: Respondents’ origins of vision problems |

# Manual axial coding results

Table S16. Manual axial coding results for blind person 1-8

| **Theme** | **Sub-theme** |
| --- | --- |
| Interviewee's Personal Information and Background | Interviewee's Vision Condition |
|  | Interviewee's Age Situation |
| Gaming Motivation |  |
|  |  |
|  | Gaming Motivation: Social Needs |
|  | Gaming Motivation: achievement |
|  | Gaming Motivation: Blind-Friendly Entertainment |
|  | Gaming Motivation: Leisure Activity |
|  | Gaming Motivation: Seeking Novel Experiences |
|  | Gaming Motivation: Fun |
| Gaming Behavior and Process | Mastery and Learning of the Game |
|  | Ways of Engaging with the Game |
|  | Frequency and Time Spent on Gaming |
|  | Issues with Using Gaming Devices |
|  |  |
|  | Challenges in the Game |
|  | Consumption and Investment in the Game |
|  | Encounters in the Game |
| Game Impact | Positive Impact of the Game |
|  | Negative Impact of the Game |
|  | Neutral Impact of the Game |
|  | Gaming experience and feelings |
|  | Suggestions for game improvement |
| Gaming Cognition and Attitude | Cognition of the Game |
|  | Attitude Towards the Game |
|  | Attraction of the Game |
| Gaming Operational Characteristics and Accessibility Design | Convenience of the Game: Auto-Pathfinding Feature |
|  | Impact of Operational Speed on Gaming Efficiency |
|  | Challenges in the Game: Level Restrictions |
|  | Accessibility Design of the Game |
|  | Assistance in the Game: Mutual Help Between High and Low-Level Players |
|  | Ease of Learning the Game: Clear Operation Prompts |
|  | Demand for Individual Strength and Compensation for Collective Advantages in the Game |
|  | Friendliness of the Game to the Visually Impaired Community |
|  | Game Mechanics: Slow Leveling Up |
|  | Simple and Easy-to-Understand Game Design |
|  | Openness and Privacy of the Game Community |
|  | Game Features: Energy-Efficient |
|  |  |
|  | Equality in the Game: No Age or Status Restrictions |
|  | Choice of Game Types and Impact of Devices |
| Gaming Social Interaction | Choice of Gaming Companions and Teams |
|  | Communities and Guilds in the Game |
|  | Social Support Provided by Game Social Features |
|  | Proportion of Gamers in the Blind Community |
|  | Social Functions of the Game |

# ChatGPT (4-Turbo) selective coding results

Here we present an example of selective coding results by ChatGPT:

First, let's perform selective coding on these categories and subcategories to determine the core category. Selective coding is a stage in qualitative research that involves identifying the category that is most relevant or most representative of the central issue or core concept of the study.

**Core Category:**

The Impact of Gaming on Life: This theme encompasses the various impacts of gaming on the lives of blind individuals, from psychological states and emotions to daily living and interpersonal relationships. This is because how gaming affects the lives of blind individuals is the central issue of this study.

**Relationships Established Around the Core Category:**

Gaming Motivation and Attitude: Why do blind people play games? How do their motivations and attitudes affect their gaming experience, and in turn, how does gaming influence their life?

Gaming Experience: There is a close relationship between the gaming experience and the impact of gaming on life. A good experience may lead to positive effects, while a poor experience could result in negative impacts.

Gaming Consumption and Investment: How does the time and money spent on gaming relate to the impact of gaming on life? For example, excessive consumption could lead to stress in life.

Gaming Operations and Learning: How does the mastery and learning of games affect the self-confidence and life satisfaction of blind individuals?

Social Interaction in Games: How does social interaction within games impact the interpersonal relationships of blind individuals and their social needs in life?

Game Accessibility Facilities and Design: How does an accessible gaming experience enhance the gaming satisfaction of blind individuals and reduce the negative impact of gaming on their life?

Personal Information and Background: How does the background information of the respondents (such as age, the origin of vision problems) interact with the impact of gaming on their lives?

In this way, we can see that each theme and subtheme is closely related to the core category (the impact of gaming on life). This provides us with a more complete and in-depth understanding of how gaming affects the lives of blind individuals.

#
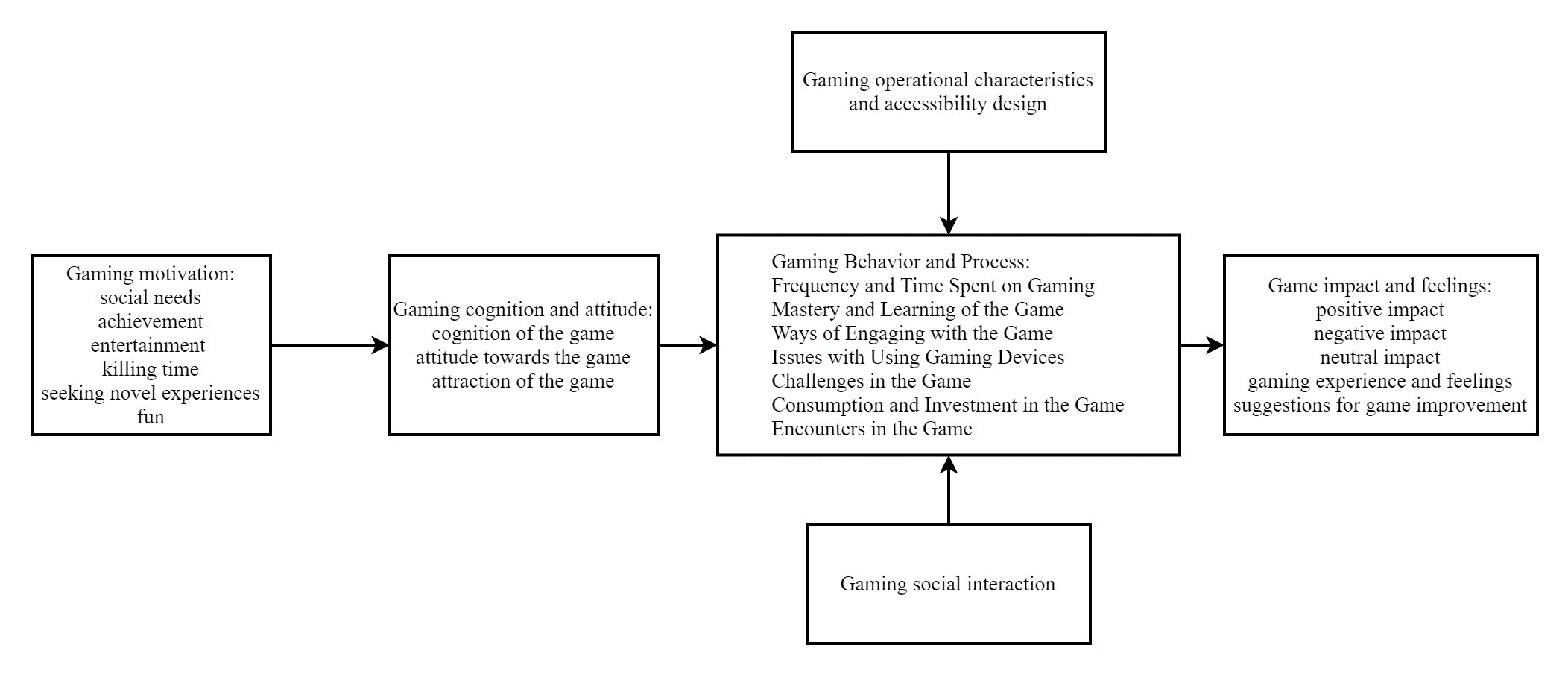
Theoretical model coded by manual coders

Fig. 1. The coding structure by manual coders: gaming behavior and impact

# Theoretical model coded by ChatGPT (4-Turbo)


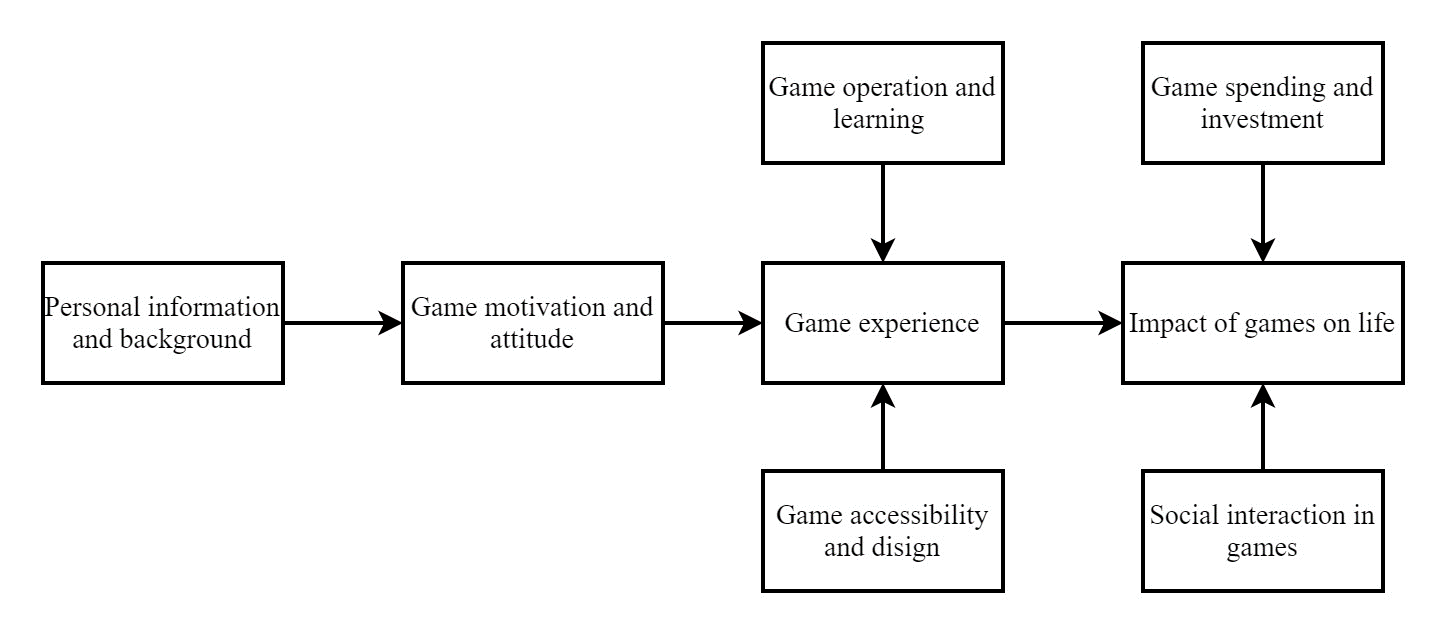


Fig. 1. The coding structure by ChatGPT: the impact of gaming on life

Note. The coding structure was developed by the researchers based on the selective coding results provided by ChatGPT, combined with their understanding and analysis of the interview text.

# VII. Generic prompts for grounded theory coding using ChatGPT (4-Turbo)

***Step 1:*** ***Setting up background prompts***

**Prompt 1.1:** You are a scholar in social sciences, skilled in using interview data and grounded theory, and proficient in coding interview texts, do you understand?

***Step 2:*** ***Performing open coding***

**Prompts for inputting longer text lengths:**

**Prompt 2.1:** This is the first part of the interview text, have you received it? You only need to remember the content of the first part and wait until all the text is sent before analyzing it.

**Prompt 2.2:** This is the second part of the interview text, have you received it?

**Prompt 2.3:** Please conduct open coding for this interview text (in two parts), and align the coding with the source in the text, presenting it in a table.

**Prompts for inputting short text lengths:**

**Prompt 2.4:** Now**,** you have received the interview text. Please conduct open coding for the interviews, and align each code with all specific statements in the text. One code can correspond to multiple statement sources. Please present the codes in a table. The table should have four columns: one for the code, one for the statement source (the sentence is required), one for the number of sources corresponding to that code, and one for the interviewees.

**To obtain more types of coding results:**

**Prompt 2.5:** The coding above is not detailed enough, please delve deeper and identify more codes.

***Step 3:*** ***Performing axial coding***

**Prompt 3.1:** Here are the results of open coding for interview text. Can you compare the similarities and differences in all the encodings of interview text, and identify the themes they share as well as their unique themes?

**Prompt 3.2:** Can you categorize all encodings for interview text under appropriate themes? Each encoding can fall under multiple themes, and each theme might have sub-themes. Please list all theme classifications.

**Prompt 3.3:** Could you illustrate the relationships between these themes and their specific encodings using a textual diagram?

**To identify more themes:**

**Prompt 3.4:** Please cluster all open coding nodes from the interview text.

**Prompt 3.5:** I’d like you to forget the previous thematic analysis you’ve done. Using all open encodings from the interview text, perform clustering again.

***Step 4:*** ***Performing selective coding***

**Prompt 4.1:** Please perform selective coding based on the following coded main categories and subcategories, identify the core category, and establish relationships around the core category.
